# Supplementary material for: KDM2B regulates hippocampal morphogenesis by transcriptionally silencing Wnt signaling in neural progenitors
Source: Nat Commun. 2023 Oct 14;14:6489. doi: 10.1038/s41467-023-42322-2 (PMC10576813; doi:10.1038/s41467-023-42322-2)
Supplement: Supplementary file 1 — Supplementary Information [file 41467_2023_42322_MOESM1_ESM.pdf]

KDM2B regulates hippocampal morphogenesis by transcriptionally silencing Wnt signaling in neural progenitors

Supplementary Information

Supplementary information contains 11 supplementary Figures, 4 supplementary tables, supplementary methods, source data and supplementary references.

Supplementary Figures

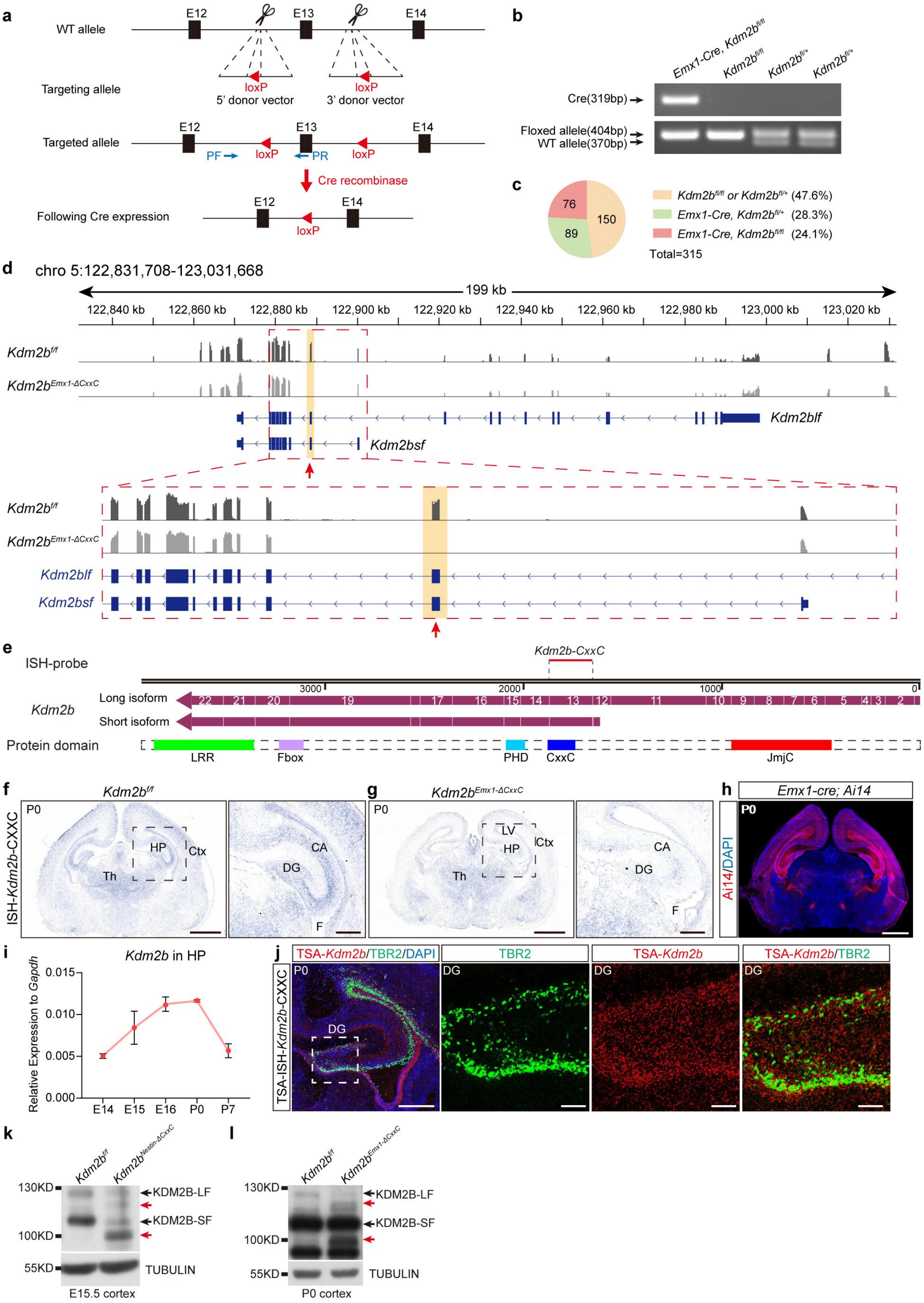

Supplementary Figure 1

Supplementary Fig. 1: Selective ablation of the KDM2B-CxxC in the developing *Kdm2b*$^{Emx1\text{-}\Delta CxxC}$ hippocampi.

a Schematic representation of the *Kdm2b* genomic structure (top), targeting allele (middle) and targeted allele (bottom). Exon 13 is flanked by two *loxP*

sites and will be excised after mating with Cre-recombinase-expressing mice. Floxed genotype primers (PF and PR) were marked with blue arrows.

b PCR products for respective genotypes. Uncropped blots in Source data.

c Offspring distribution of indicated genotypes at P0 from *Kdm2b*$^{fl/fl}$ females crossing with *Emx1-Cre*; *Kdm2b*$^{fl/+}$ males.

d RNA-seq track of P0 control and *Kdm2b*$^{Emx1\text{-}\Delta CxxC}$ hippocampal neurospheres showed deletion of exon 13 (the red arrow).

e Schematic diagram of *Kdm2b* transcripts and corresponding protein domains. Positions of the ISH probe was indicated.

f, **g** Representative ISH images showing *Kdm2b* expression in coronal sections of P0 control (**f**) and *Kdm2b*$^{Emx1\text{-}\Delta CxxC}$ (**g**) brains. Hippocampi (HP) were enlarged on the right.

h Representative immunofluorescent image of P0 *Emx1-cre*;*Ai14* brain. Nuclei were labeled with DAPI (blue).

i Relative expression of *Kdm2b* in hippocampus at different stages of development.

j Representative ISH-fluorescence staining of *Kdm2b* and immunofluorescence co-staining of TBR2 in the coronal section of P0 wild-type mouse brains. Boxed regions of DG were enlarged on the right.

k, **l** Immunoblots of KDM2B and TUBLIN using extracts of E15.5 control and *Kdm2b*$^{Nestin\text{-}\Delta CxxC}$ neocortices (**k**), and P0 control and *Kdm2b*$^{Emx1\text{-}\Delta CxxC}$ neocortices (**l**). Black arrows indicated wild-type KDM2B, whereas red arrows indicated CxxC-ZF deleted KDM2B. Uncropped blots in Source data.

Scale bars, 1mm (**f**, **g** left, **h**), 300 μm (**f**, **g** right, **j** left), 50 μm (**j** right). HP, Hippocampus; Ctx, cortex; Th, Thalamus; CA, Cornu Ammonis; DG, dentate gyrus; LV, lateral ventricle; F, fimbria.

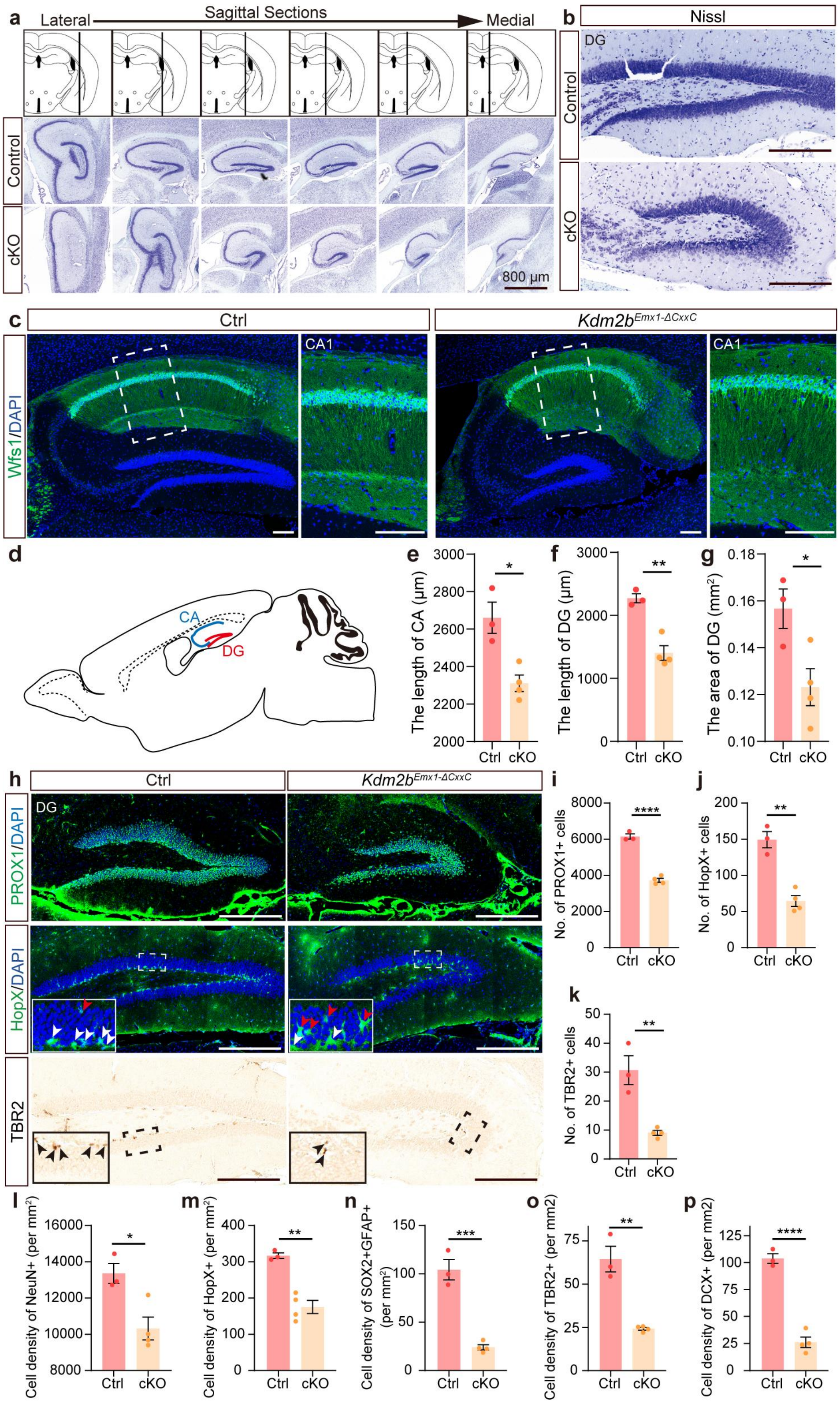

Supplementary Figure 2

Supplementary Fig. 2. Deletion of the KDM2B-CxxC causes hippocampal hypoplasia.

a Nissl staining of sagittal sections of adult control and *Kdm2b*$^{Emx1\text{-}\Delta CxxC}$

hippocampi. Sections from lateral to medial were displayed sequentially from left to right, with relative positions shown as black lines on the first line.

b Nissl staining on sagittal sections of adult control (left) and *Kdm2b*$^{Emx1\text{-}\Delta CxxC}$ (right) DGs.

c Immunofluorescent (IF) staining of Wfs1 on sagittal sections of adult control (left) and *Kdm2b*$^{Emx1\text{-}\Delta CxxC}$ (right) hippocampi. Nuclei were labeled with DAPI (blue). Boxed regions of CA1 were enlarged on the right.

d The schematic diagram of a sagittal section of adult brain. The DG and CA regions were marked by red and blue lines, respectively.

e-**g** Quantifications of the length of CA (**e**), the length of DG (**f**), and the area of DG (**g**) in adult control and *Kdm2b*$^{Emx1\text{-}\Delta CxxC}$ brain.

h From top to bottom: IF staining for PROX1, IF for HopX and IHC for TBR2, on sagittal sections of adult control (left) and *Kdm2b*$^{Emx1\text{-}\Delta CxxC}$ (right) DGs. Boxed regions of SGZ were enlarged on the left-bottom corners. Boxed regions were enlarged on bottom-left corners. White and red arrows denote HopX+ signals in the subgranule zone (SGZ) and granule cell layer respectively.

i-**k** Quantification of the number of PROX1+ cells (**i**), HopX+ cells (**j**) and DCX+ cells (**k**) in the DG.

l-**p** Quantification of the density of NeuN+ cells in the DG (**l**), HopX+ cells in the SGZ (**m**) and SOX2+GFAP+ cells in the SGZ (**n**), TBR2+ cells in the SGZ (**o**), DCX+ cells in the DG (**p**).

n = 3 for control brains and n = 4 for *Kdm2b*$^{Emx1\text{-}\Delta CxxC}$ brains in (**e**-**g**, **i**-**p**). Data are represented as means ± SEM. Statistical significance was determined using an unpaired two-tailed Student's t-test (**e**-**g**, **i**-**p**). *P < 0.05, **P < 0.01, ***P < 0.001, and ****P < 0.0001. Scale bars, 800 μm (**a**), 200 μm (**b**, **h**), 100 μm (**c**).

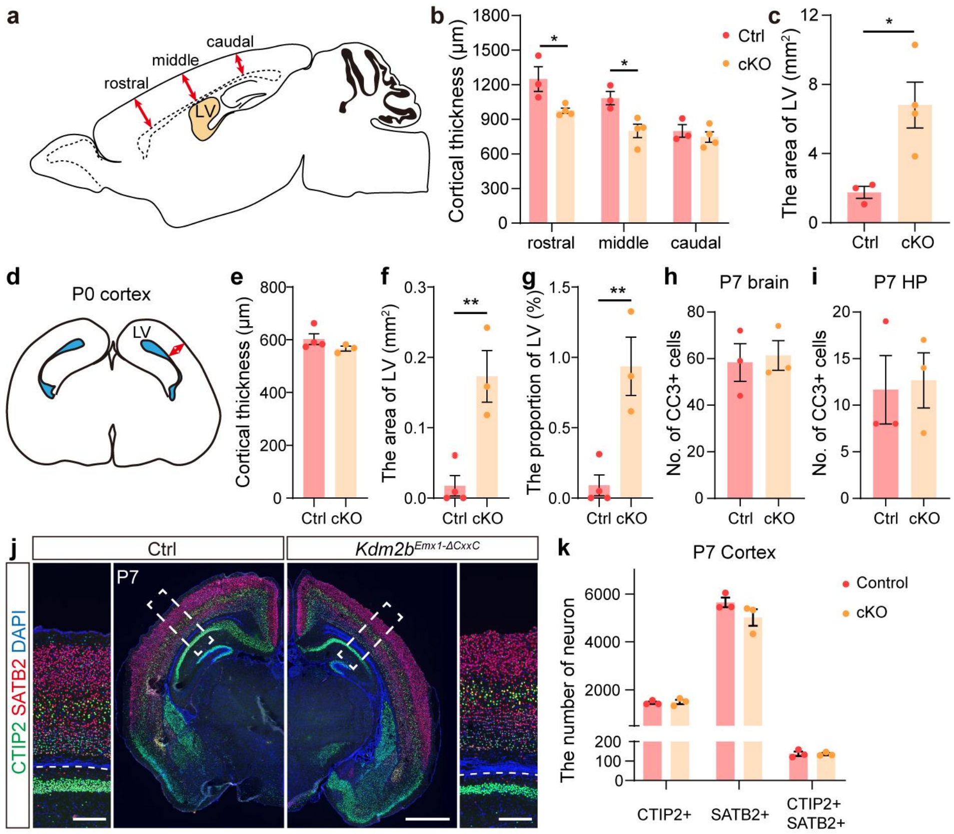

Supplementary Figure 3

Supplementary Fig. 3. Neocortical development was unaffected on conditional loss of KDM2B-CxxC.

a The schematic diagram of a sagittal section of adult brain. Red bidirectional arrows show the rostral, middle, and caudal regions of the neocortex.

b Quantifications of neocortical thickness of adult control and $Kdm2b^{Emx1\text{-}\Delta CxxC}$ brains in rostral, middle, and caudal regions.

c Quantifications of the area of lateral ventricle in adult control and $Kdm2b^{Emx1\text{-}\Delta CxxC}$ brain. n = 3 for control brains and n = 4 for $Kdm2b^{Emx1\text{-}\Delta CxxC}$ brains.

d The diagram of a coronal section of P0 brain. The red bidirectional arrow indicates neocortical thickness. The blue areas indicate the lateral ventricles (LV).

e-g Quantifications of neocortical thickness (**e**), area of lateral ventricles (LV, **f**) and the area proportion of LV (**g**) in P0 control and $Kdm2b^{Emx1\text{-}\Delta CxxC}$ brains. n = 4 for control brains and n = 3 for $Kdm2b^{Emx1\text{-}\Delta CxxC}$ brains.

h-**i** Quantifications of cleaved Caspase3 (CC3) labeled apoptotic cells in the coronal section (**h**) and HP region (**i**) of P7 brain.

j IF staining for CTIP2 (green) and SATB2 (red) on coronal sections of P7 control (left) and *Kdm2b*$^{Emx1\text{-}\Delta CxxC}$ (right) mice. Nuclei were labeled with DAPI (blue). Boxed regions were enlarged on the right.

k Quantification of CTIP2+, SATB2+ and CTIP2+SATB2+ cells in P7 cortex (**j**). n = 3 for control brains and n = 3 for *Kdm2b*$^{Emx1\text{-}\Delta CxxC}$ brains.

Data are represented as means ± SEM. Statistical significance was determined using two-way ANOVA followed by Sidak's multiple comparisons test (**b**, **k**), or using an unpaired two-tailed Student's t-test (**c**, **e**-**i**). *P < 0.05, **P < 0.01. Scale bars, 1 mm (**j**, whole brain), 200 μm (**j**, cortex). LV, lateral ventricles.

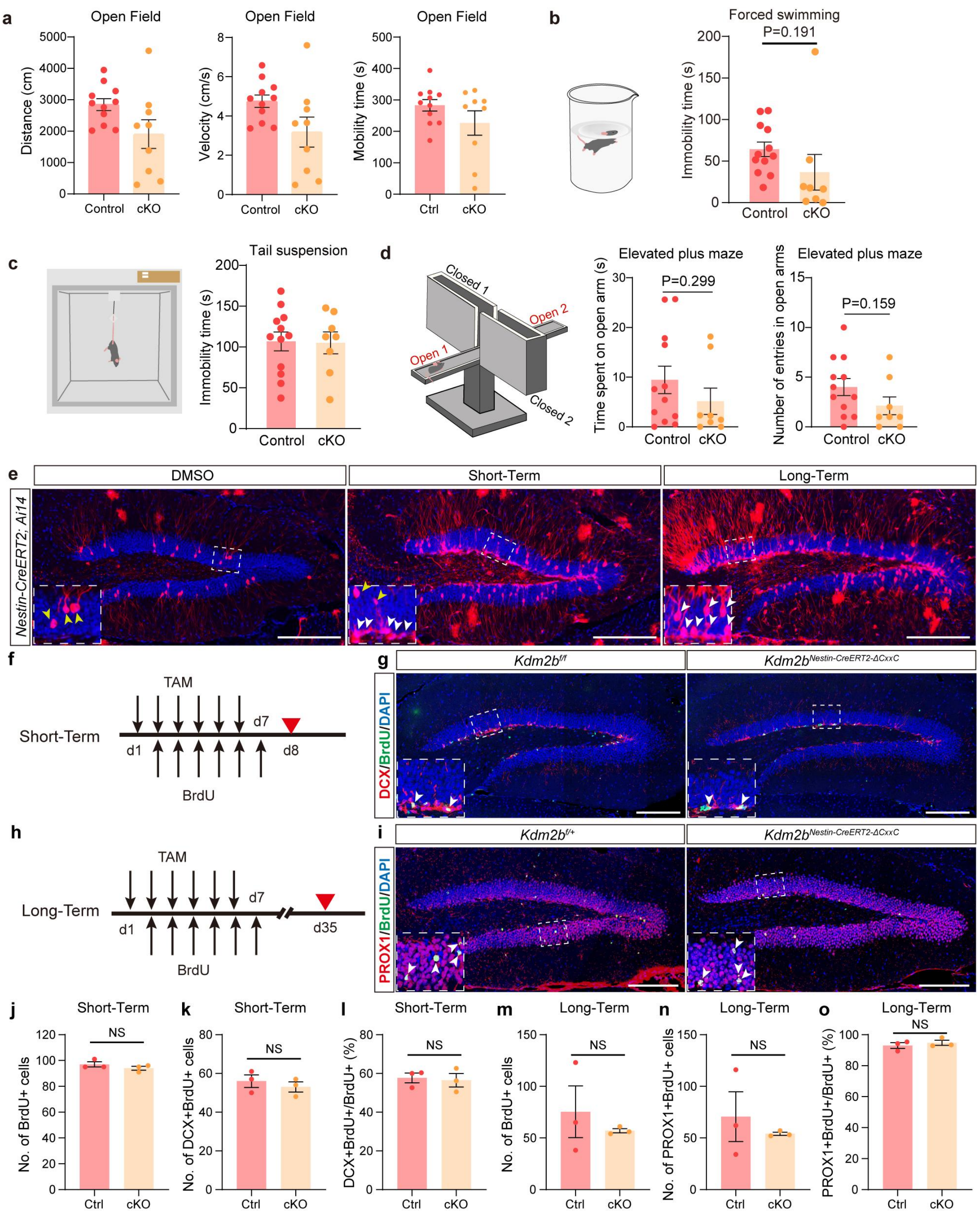

Supplementary Figure 4

Supplementary Fig. 4. SGZ neurogenesis was unaffected on ablation of KDM2B-CxxC at the adult stage.

a Quantification of distance, velocity, and mobility time in the open-field test.

b, **c** Immobility time in forced swimming (**b**) and tail suspension experiments (**c**).

d Time spent in open arms and the number of entries in open arms in elevated

plus maze test.

e Representative IF images showing DG region of *Nestin-CreERT2; Ai14* mice intraperitoneal injected with DMSO, short-term TAM and long-term TAM. Nuclei were labeled with DAPI (blue). Boxed regions were enlarged on bottom-left corners. White arrows denote Nestin+ cells and their progeny in DG.

f, **h** Eight-week-old mice ($Kdm2b^{f/f}$ or $Kdm2b^{f/+}$, and $Kdm2b^{Nestin\text{-}CreERT2\text{-}\Delta CxxC}$) were intraperitoneally injected with TAM and BrdU for 6 consecutive days (BrdU injection delayed by 1 day). Mice were sacrificed either 1 day later (**f**, Short-Term) or 4 weeks later (**h**, Long-Term).

g Representative IF images showing DG sections stained with DCX (red) and BrdU (green). Nuclei were labeled with DAPI (blue). Boxed regions were enlarged on bottom-left corners. White arrows denote DCX+BrdU+ cells in DG.

i Representative IF images showing DG sections stained with PROX1 (red) and BrdU (green). Nuclei were labeled with DAPI (blue). Boxed regions were enlarged on bottom-left corners. White arrows denote PROX1+BrdU+ cells in DG.

j-**l** Quantification of numbers of BrdU+ cells (**j**), DCX+BrdU+ cells (**k**), and the proportion of DCX+BrdU+/BrdU+ (**l**) in Short-Term experiments.

m-**o** Quantification of numbers of BrdU+ cells (**m**), PROX1+BrdU+ cells (**n**), and the proportion of PROX1+BrdU+/BrdU+ (**o**) in Long-Term experiments.

In (**a**), n = 11 mice for control and n = 9 mice for $Kdm2b^{Emx1\text{-}\Delta CxxC}$. In (**b**-**d**), n = 12 mice for control and n = 8 mice for $Kdm2b^{Emx1\text{-}\Delta CxxC}$. In (**f**-**o**), n = 3 mice for both control and for $Kdm2b^{Nestin\text{-}CreERT2\text{-}\Delta CxxC}$. Data are represented as means ± SEM. Statistical significance was determined using an unpaired two-tailed Student's t-test (**a**-**d**, **j**-**o**). Scale bars, 200 μm (**e**, **g**, **i**).

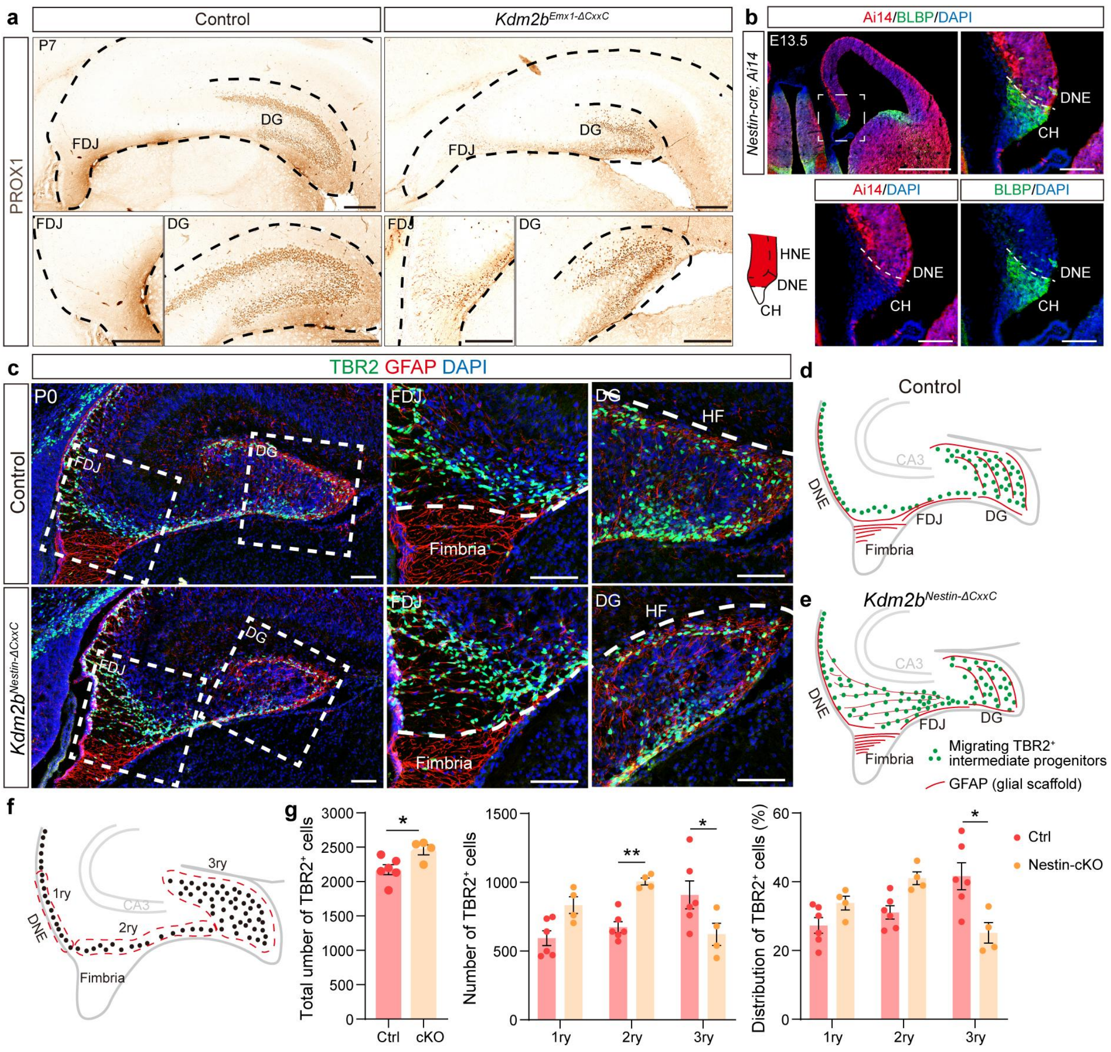

Supplementary Figure 5

Supplementary Fig. 5. Ablation of the KDM2B-CxxC blocked the migration of intermediate progenitors and neurogenesis of granule cells.

a Immunohistochemical staining for PROX1 on coronal sections of P7 control (left) and *Kdm2b*$^{Emx1\text{-}\Delta CxxC}$ (right) hippocampi. The DG and FDJ regions were individually enlarged underneath.

b IF staining for BLBP (green) on E13.5 *Nestin-cre*;*Ai14* brain sections. Nuclei were labeled with DAPI (blue). Boxed regions are enlarged and single channel fluorescence staining of Ai14 (tdTomato) and BLBP were shown respectively. Dashed lines separate DNE and CH. The schematic diagram of Nestin expression is shown at the bottom left.

c Double immunofluorescence of TBR2 (green) and GFAP (red) on P0 wild-type and *Kdm2b*$^{Nestin\text{-}\Delta CxxC}$ hippocampi. Nuclei were labeled with DAPI

(blue). Boxed regions of FDJ and DG were enlarged on the right.

d, **e** The schematic of P0 control and *Kdm2b*$^{Nestin\text{-}\Delta CxxC}$ hippocampi. Green dots represent migrating TBR2+ intermediate progenitors, and red lines represent GFAP+ glial scaffold.

f, **g** Distribution of TBR2+ cells along the three matrices, where dashed lines indicate areas considered as the 1ry, 2ry, and 3ry matrix (**f**).

Cartoons in **d**, **e** and **f** are adapted from Caramello *et al.*[1]. n = 6 for control brains and n = 4 for *Kdm2b*$^{Nestin\text{-}\Delta CxxC}$ brains. Data are represented as means ± SEM. Statistical significance was determined using an unpaired two-tailed Student's t-test (**g** left), or using two-way ANOVA followed by Sidak's multiple comparisons test (**g** middle and right). *P < 0.05, **P < 0.01. Scale bars, 200 µm (**a**), 500 µm (**b**, upper-left), 100 µm (**a** enlarged box regions, **c**). DG, dentate gyrus; DMS, dentate migratory stream; FDJ, fimbriodentate junction; HF, hippocampal fissure; 1ry, primary matrix; 2ry, secondary matrix; 3ry, tertiary matrix; CH, cortical hem; HNE, hippocampal neuroepithelium; DNE, dentate neuroepithelium.

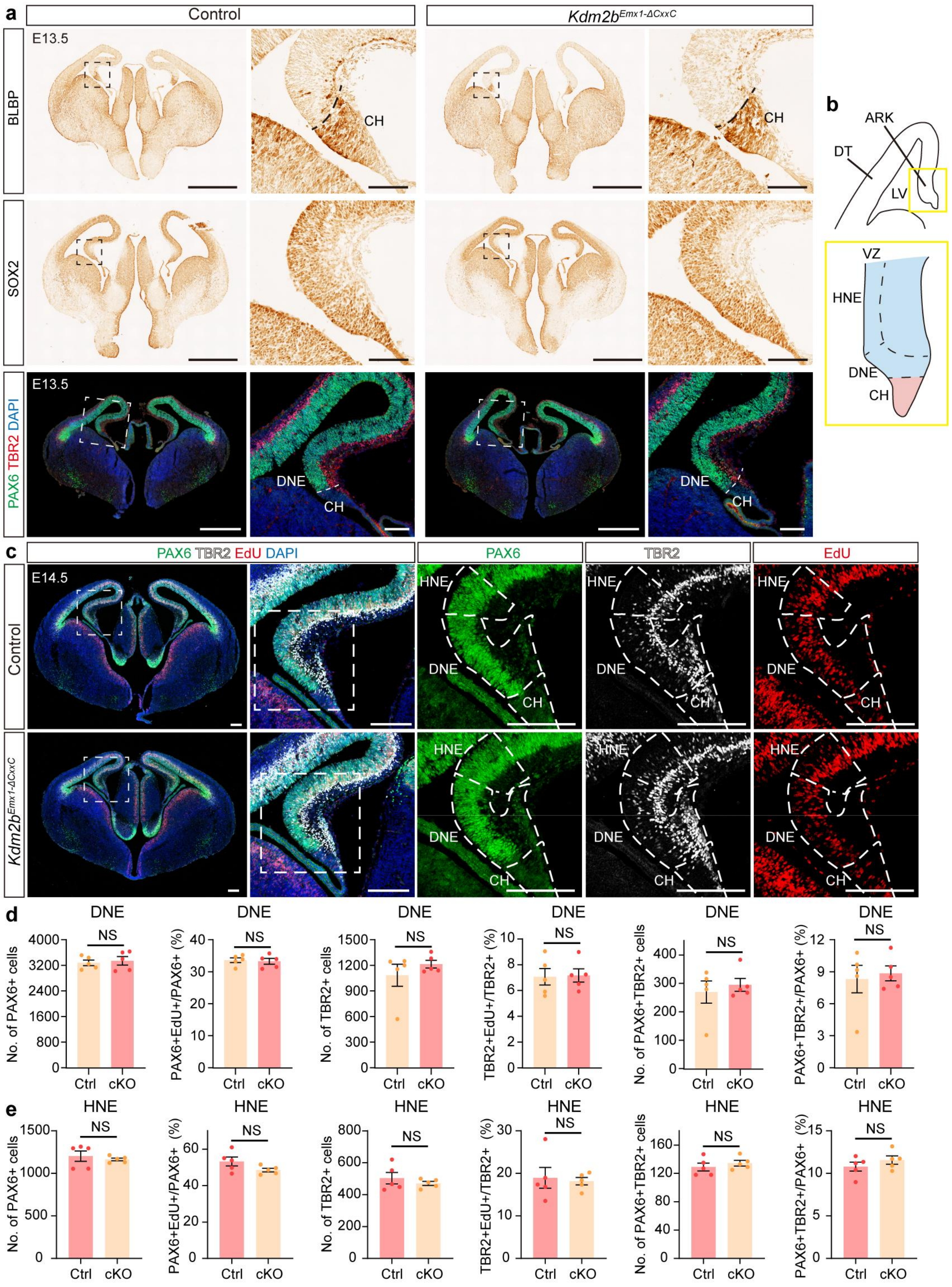

Supplementary Figure 6

Supplementary Fig. 6. Unaltered neural progenitor domains in *Kdm2b*$^{Emx1\text{-}\Delta CxxC}$ hippocampal primordia.

a Immunohistochemical staining for BLBP (top) and SOX2 (middle). Bottom, immunofluorescent staining for PAX6 (green) and TBR2 (green) on coronal sections of E13.5 control (left) and *Kdm2b*$^{Emx1\text{-}\Delta CxxC}$ (right) brains. Nuclei were

labeled with DAPI (blue). Boxed regions were enlarged on the right.

b The schematic of E13.5 wild-type hippocampus. The yellow box is enlarged to show the hippocampal primordia, with dashed lines demarcating HNE, DNE and CH.

c Triple-labeling of PAX6 (green), TBR2 (white) and EdU (red) on E14.5 control and *Kdm2b*$^{Emx1\text{-}\Delta CxxC}$ brain sections. Pregnant mice were injected with EdU 2 h before sacrifice. Nuclei were labeled with DAPI (blue). Boxed regions are enlarged on the right, and single channel fluorescence staining of PAX6, TBR2 and EdU were shown respectively. Dashed lines indicate HNE, DNE and CH.

d, **e** Quantification of data in (**c**). Quantification of number of PAX6+, TBR2+, PAX6+TBR2+ and TBR2+EdU+ cells, and the proportion of PAX6+EdU+/PAX6+, TBR2+EdU+/TBR2+ and PAX6+TBR2+/PAX6+ in E14.5 control and *Kdm2b*$^{Emx1\text{-}\Delta CxxC}$ DNE (**d**) and HNE (**e**).

Cartoons in **b** are adapted from Caramello *et al.*[1]. n = 5 for control brains and n = 5 for *Kdm2b*$^{Emx1\text{-}\Delta CxxC}$ brains. Data are represented as means ± SEM. Statistical significance was determined using an unpaired two-tailed Student's t-test (**d**, **e**). Scale bars, 1 mm (**a**, whole brain), 100 μm (**a**, HP), 200 μm (**c**). CH, cortical hem; LV, lateral ventricle; DT, dorsal telencephalon; ARK, archicortex; HNE, hippocampal neuroepithelium; DNE, dentate neuroepithelium; VZ, ventricular zone.

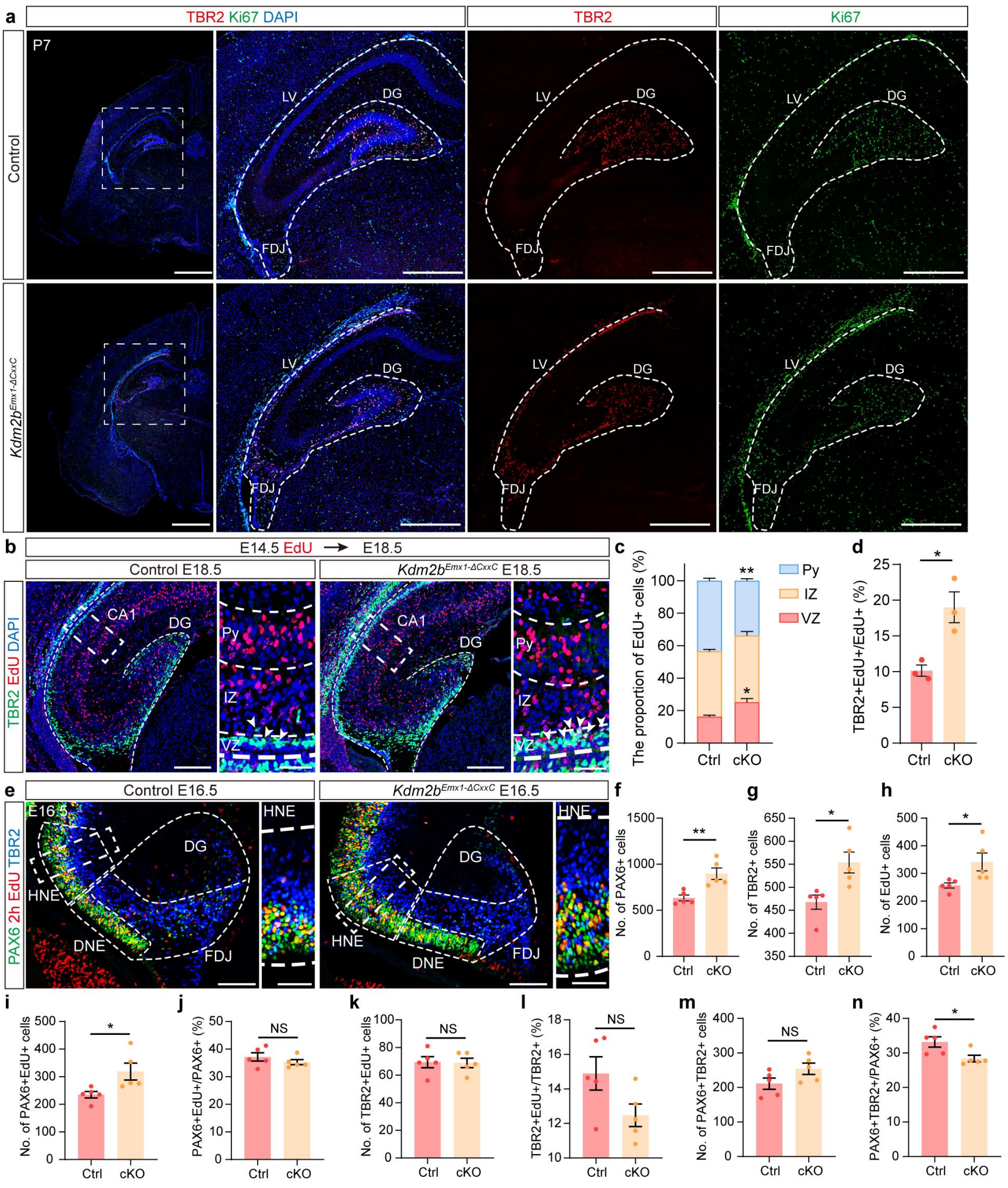

Supplementary Figure 7

Supplementary Fig. 7. Ablation of the KDM2B-CxxC blocked neurogenesis in the CA region.

a Double-labeling of TBR2 (red) and Ki67 (green) on P7 coronal section of control and *Kdm2b*$^{Emx1\text{-}\Delta CxxC}$ brains. Nuclei were labeled with DAPI (blue). Boxed regions are enlarged on the right, and single channel fluorescence staining of TBR2 and Ki67 were shown respectively. Dashed lines outline the hippocampi.

b EdU was administrated at E14.5 and double labeling of TBR2 and EdU was

performed on E18.5 coronal sections. Boxed regions were enlarged on the right. Dashed lines outline the hippocampi and distinguish three layers of CA1: VZ, IZ, Py.

c The relative location of EdU+ cells in VZ, IZ, Py were quantified.

d Quantification of the proportion of TBR2+EdU+/EdU+ in control and *Kdm2b*$^{Emx1\text{-}\Delta CxxC}$ CA1.

e Triple-labeling of PAX6 (green), TBR2 (blue) and EdU (red, 2h labeling) on E16.5 control and *Kdm2b*$^{Emx1\text{-}\Delta CxxC}$ brain sections. Boxed regions (HNE) were enlarged on the right. Dashed lines outline the hippocampi and distinguish HNE, DNE, FDJ and DG.

f-**h** Quantification of PAX6+ (**f**), TBR2+ (**g**) and EdU+ (**h**) cells in the HNE.

i, **k**, **m** Quantification of PAX6+EdU+ (**i**), TBR2+EdU+ (**k**) and PAX6+TBR2+ (**m**) cells in the HNE.

j, **l**, **n** Quantification of the proportion of PAX6+EdU+/PAX6+ (**j**), TBR2+EdU+/TBR2+ (**l**) and PAX6+TBR2+/PAX6+ (**n**) in the HNE.

n = 3 for control brains and n = 3 for *Kdm2b*$^{Emx1\text{-}\Delta CxxC}$ brains in (**c**, **d**). n = 5 for control brains and n = 5 for *Kdm2b*$^{Emx1\text{-}\Delta CxxC}$ brains in (**f**-**n**).

Data are represented as means ± SEM. Statistical significance was determined using an unpaired two-tailed Student's t-test (**d**, **f**-**n**), or using two-way ANOVA followed by by Tukey's multiple comparisons test (**c**). *P < 0.05, **P < 0.01, NS, not significant. Scale bars, 1 mm (**a**, whole brain), 100 μm (**a**, HP), 200 μm (**b** left, **e** left), 50 μm (**b** right), 40 μm (**e** right). DG, dentate gyrus; VZ, ventricular zone; IZ, intermediated zone; Py, pyramidal cell layer of the hippocampus; HNE, hippocampal neuroepithelium; DNE, dentate neuroepithelium; FDJ, fimbriodentate junction.

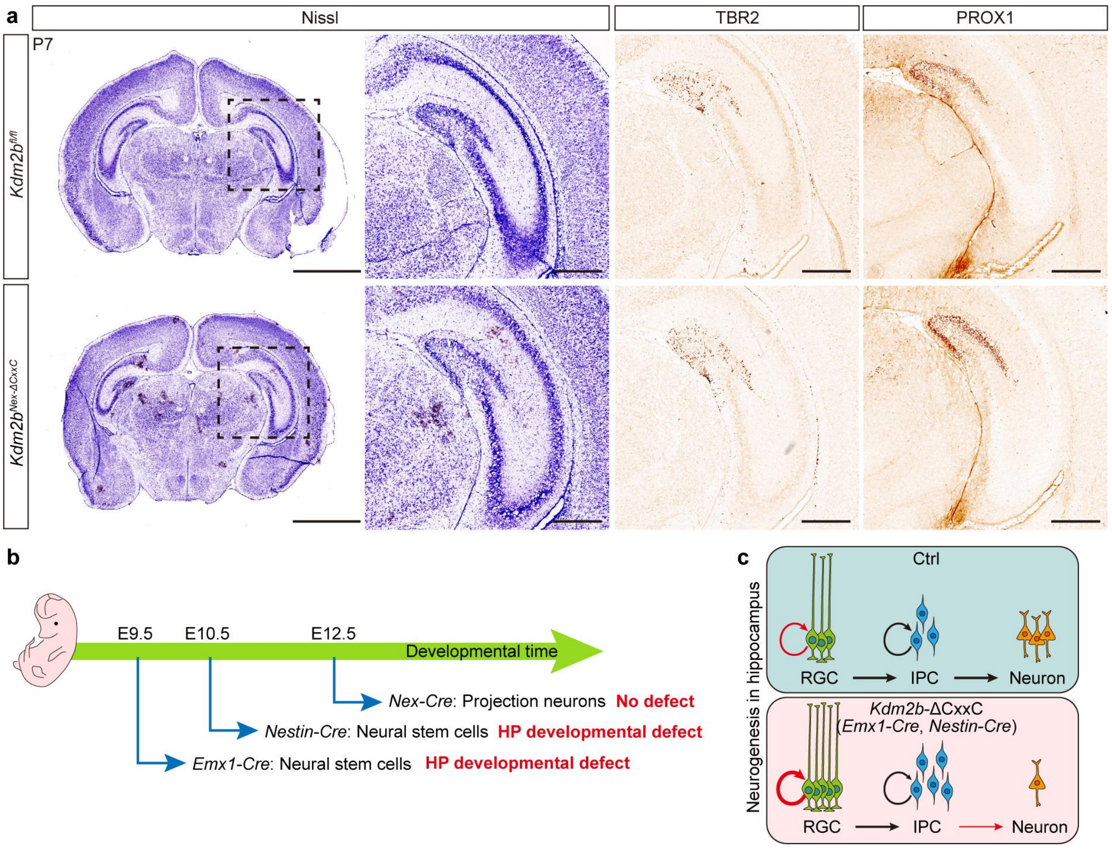

Supplementary Figure 8

Supplementary Fig. 8. Abnormal neuronal differentiation is not responsible for hippocampal hypoplasia caused by loss of KDM2B-CxxC.

a Nissl staining, and immunohistochemical staining of TBR2 and PROX1 on coronal sections of P7 control and *Kdm2b*$^{Nex\text{-}\Delta CxxC}$ brains.

b The schematic diagram summarizing expression profiles of three Cre lines, as well as hippocampal phenotypes of respective cKO mice.

c The diagram showing aberrant neurogenesis during hippocampal development of *Kdm2b*$^{Emx1\text{-}\Delta CxxC}$ and *Kdm2b*$^{Nestin\text{-}\Delta CxxC}$ mice: a bigger RGC pool with more IPCs produced, but differentiation of IPCs toward neurons was compromised.

Scale bars, 2 mm (**a**, whole brain), 500 μm (**a**, HP). LV, lateral ventricle; FDJ, fimbriodentate junction; DG, Dentate gyrus; RGC, Radial glia cells; IPC, Intermediate progenitor cell.

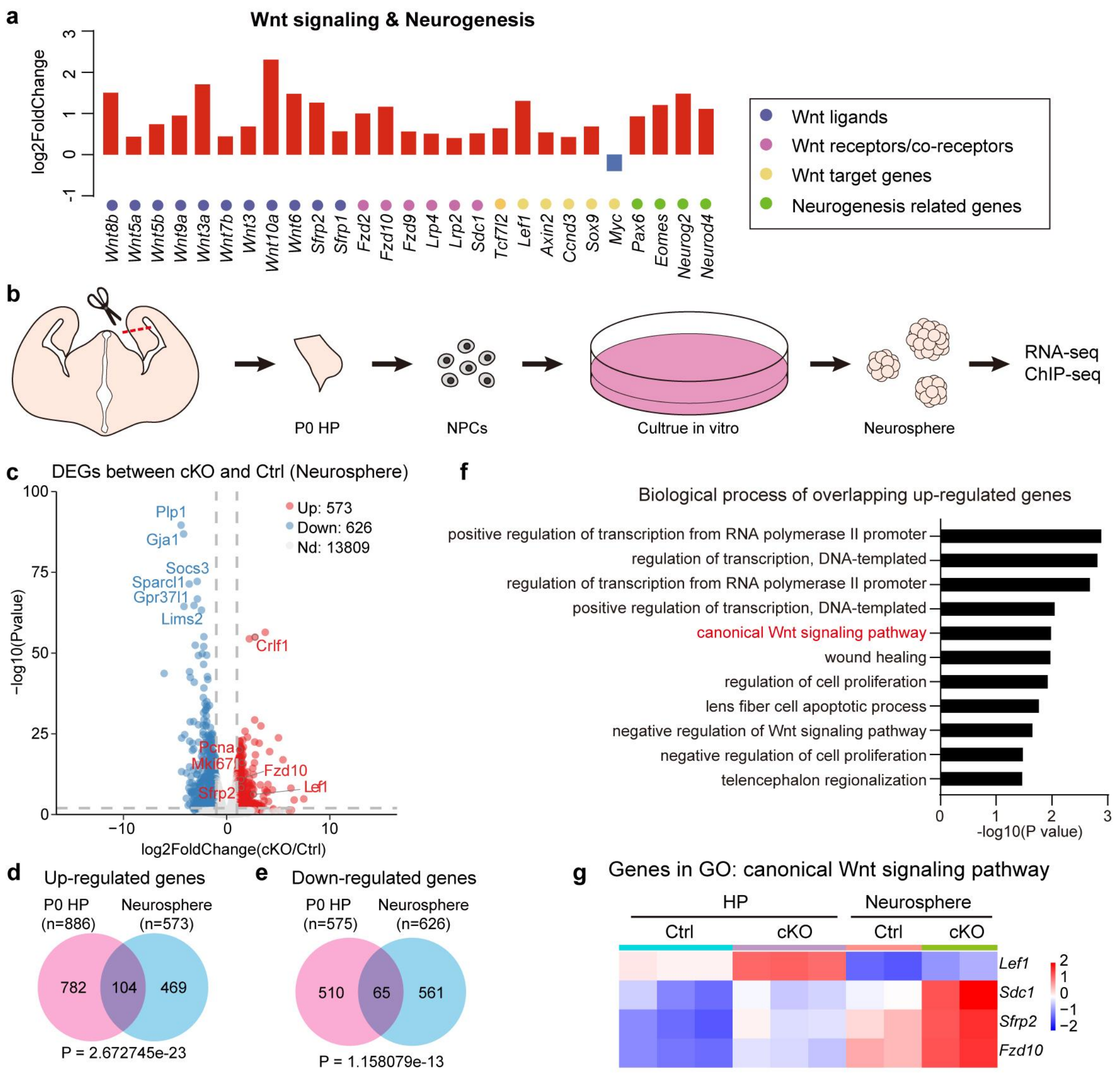

Supplementary Figure 9

Supplementary Fig. 9. RNA-seq of neurospheres derived from P0 cKO hippocampi showed activation of the Wnt signaling pathway.

a Histograms showing the log-fold change of significantly up- or down-regulated genes in *Kdm2b*$^{Emx1\text{-}\Delta CxxC}$ (cKO) hippocampi.

b The schematic diagram: hippocampal tissue was collected from P0 brain and digested into single-cell suspensions. After cultured *in vitro* for three generations, neurospheres were subjected to RNA-seq and ChIP-seq analyses.

c The volcano plot of genes up-regulated (red) and down-regulated (blue) in P0 *Kdm2b*$^{Emx1\text{-}\Delta CxxC}$ (cKO) neurospheres compared to controls.

d Overlapping up-regulated genes (104) of P0 HP and neurospheres.

e Overlapping down-regulated genes (65) of P0 HP and neurospheres.

f GO analysis of the biological process of overlapping up-regulated genes in *Kdm2b*$^{Emx1\text{-}\Delta CxxC}$ (cKO) neurospheres revealed terms related to canonical Wnt signaling pathways (red).

g The heat map of genes in GO in (**f**): canonical Wnt signaling pathway of P0 HP and neurospheres.

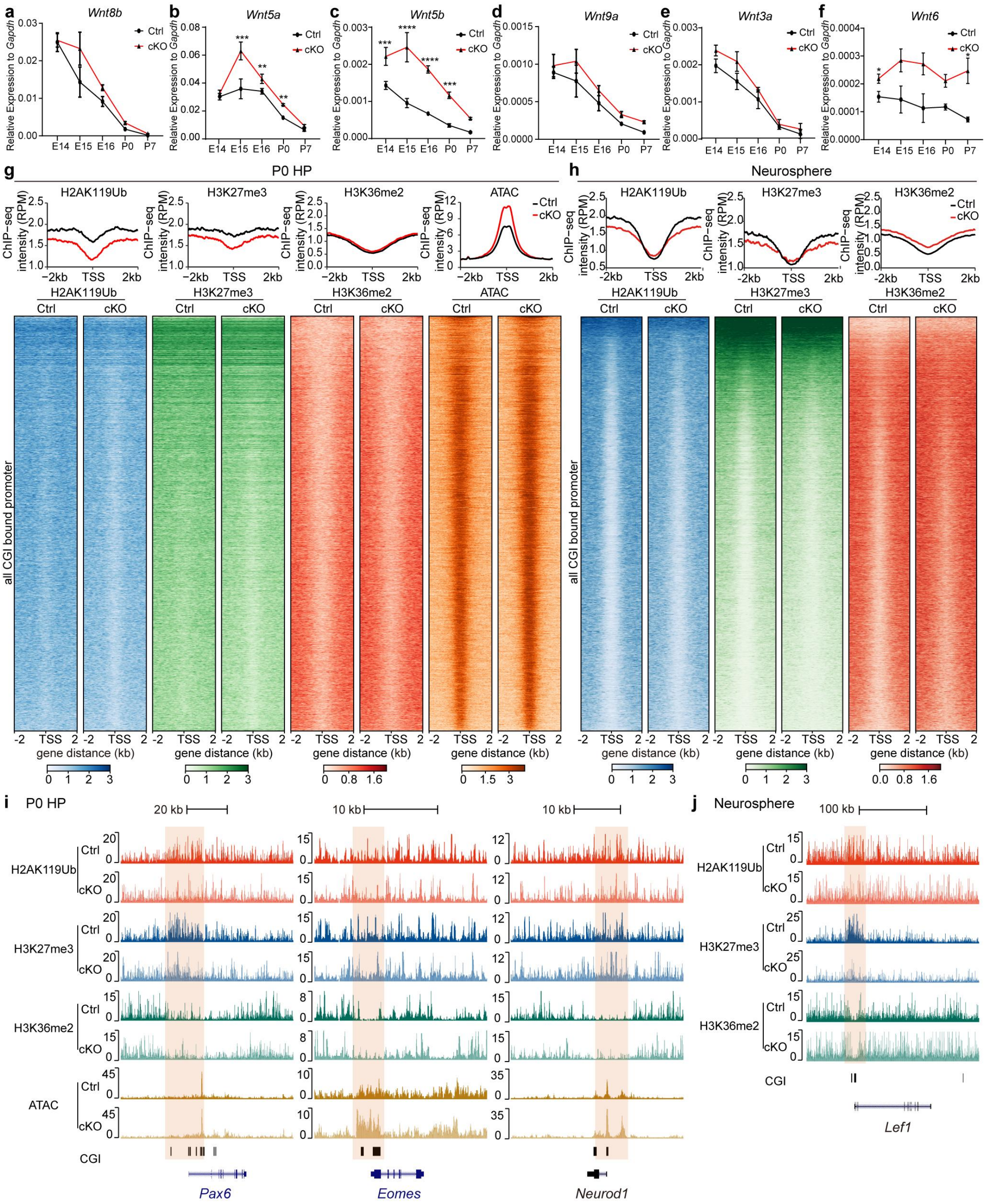

Supplementary Figure 10

Supplementary Fig. 10. KDM2B epigenetically silences components of Wnt signaling genes in developing hippocampi.

a-**f** RT-qPCR showing relative expressions of *Wnt8b*, *Wnt5a*, *Wnt5b*, *Wnt9a*, *Wnt3a* and *Wnt6* in control (black lines) and *Kdm2b*$^{Emx1\text{-}\Delta CxxC}$ (red lines) hippocampi of indicated developmental stages (E14.5, E15.5, E16.5, P0 and P7).

g Line charts (top) and heatmaps (bottom) showing H2AK119ub, H3K27me3,

H3K36me2, and ATAC-seq signals in control and *Kdm2b*$^{Emx1\text{-}\Delta CxxC}$ (cKO) hippocampi at all CGI-associated promoters. Colors represent ChIP-seq RPM (reads per million), and rows were ranked by ChIP-seq signals in control H2AK119ub. Line charts on the top of each set of heatmap showing average signals, with control in black and *Kdm2b*$^{Emx1\text{-}\Delta CxxC}$ (cKO) in red.

h Line charts (top) and heatmaps (bottom) showing H2AK119ub, H3K27me3 and H3K36me2 signals in control and *Kdm2b*$^{Emx1\text{-}\Delta CxxC}$ (cKO) neurospheres at all CGI-associated promoters.

i The UCSC genome browser view of H2AK119ub, H3K27me3 and H3K36me2 enrichment and ATAC-seq signal in P0 control and *Kdm2b*$^{Emx1\text{-}\Delta CxxC}$ (cKO) hippocampi at *Pax6*, *Eomes* and *Neurod1*. CGIs were shown as black columns on the bottom. Colored regions marked enrichment differences between control and cKO.

j The UCSC genome browser view of H2AK119ub, H3K27me3 and H3K36me2 enrichment in control and *Kdm2b*$^{Emx1\text{-}\Delta CxxC}$ (cKO) neurospheres at *Lef1*. CGIs were shown as black columns on the bottom. Colored regions marked enrichment differences between control and cKO.

Data are represented as means ± SEM. Statistical significance was determined using two-way ANOVA followed by Sidak's multiple comparisons test (**a**-**f**). *P < 0.05, **P < 0.01, ***P < 0.001, and ****P < 0.0001.

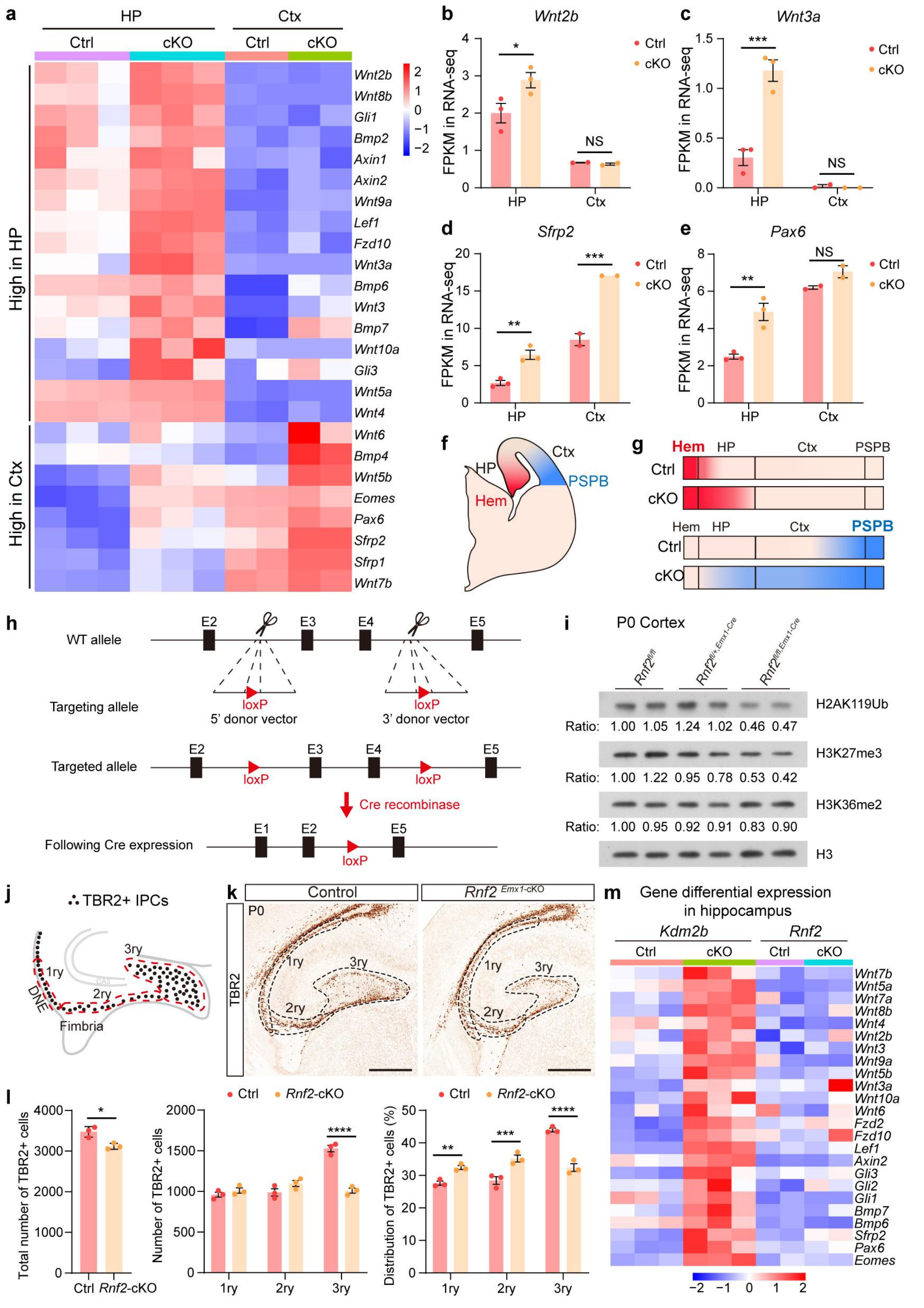

Supplementary Figure 11

Supplementary Fig. 11. Loss of Ring1B did not cause accumulation of neural progenitors.

a The heat map of hem and PSPB (pallial-subpallial boundary) genes of P0 HP

and cortex.

b-**e** Representative FPKM value of RNA-seq in (**a**): *Wnt2b* (**b**); *Wnt3b* (**c**); *Sfrp2* (**d**); *Pax6* (**e**).

f, **g** Schematic diagram of altered hem and PSPB signals in P0 *Kdm2b*$^{Emx1\text{-}\Delta CxxC}$ (cKO) brain.

h Schematic representation of the *Rnf2* genomic structure (top), targeting allele (middle) and targeted allele (bottom). Exon 3-4 is flanked by *loxP* sites and will be excised after mating with Cre-recombinase-expressing mice.

i Immunoblots of H2AK119ub, H3K27me3, H3K36me2 and H3 using extracts of P0 *Rnf2*$^{fl/fl}$, *Rnf2*$^{fl/+,\ Emx1\text{-}Cre}$ and *Rnf2*$^{fl/fl,\ Emx1\text{-}Cre}$ neocortices. The relative expression levels of indicated modifications were shown under each blot.

j-**l** Immunohistochemical staining of TBR2 (**k**) on coronal sections of P0 control (left) and *Rnf2*$^{Emx1\text{-}cKO}$ (right) hippocampi. Distribution of TBR2+ cells along the three matrices, where dashed lines indicate areas considered as 1ry, 2ry, and 3ry matrix (**l**). n = 3 for control brains, n = 3 for *Rnf2*$^{Emx1\text{-}cKO}$ brains.

m The heat maps of Wnt signaling and neurogenesis related genes in the hippocampus of P0 *Kdm2b*-cKO and *Rnf2*-cKO mice.

Data are represented as means ± SEM. Statistical significance was determined using an unpaired two-tailed Student's t-test (**i**, left) or using two-way ANOVA followed by Sidak's multiple comparisons test (**b**-**e**, **i**, middle and right). *P < 0.05, **P < 0.01, ***P < 0.001, ****P < 0.0001, and "NS" indicates no significance. Scale bars, 300 μm (**k**). HP, hippocampus; Ctx, cortex; PSPB, pallial-subpallial boundary; DNE, dentate neuroepithelium; 1ry, primary matrix; 2ry, secondary matrix; 3ry, tertiary matrix.

Supplementary Tables

Supplementary Table 1. Abbreviations list

HP	hippocampus
CA	Cornu Ammonis
DG	dentate gyrus
Ctx	cortex
Th	Thalamus
LV	lateral ventricle
F	fimbria
DMS	dentate migratory stream
FDJ	fimbriodentate junction
HF	hippocampal fissure
1ry	primary matrix
2ry	secondary matrix
3ry	tertiary matrix
HNE	hippocampal neuroepithelium
DNE	dentate neuroepithelium
CH	cortical hem
RGC	Radial glia cells
IPC	Intermediate progenitor cell
DT	dorsal telencephalon
ARK	archicortex
Sub	Subiculum
VZ	ventricular zone
IZ	intermediated zone
Py	pyramidal cell layer of the hippocampus
CGI	CpG island
PSPB	pallial-subpallial boundary

Supplementary Table 2. ISH primers used in this study

Kdm2b-CxxC-ISH-F	GTCGGCCTAAGGGCAAGTT
Kdm2b-CxxC-ISH-R	CACACACAAGGCACACGG
Lef1-ISH-F	AGAGAACACCCTGATGAAGGAA
Lef1-ISH-R	CTTCCTCTTCTTCTTCTTGCCA
Sfrp2-ISH-F	AGCAACTGCAAGCCCATC
Sfrp2-ISH-R	ATGGAGAGAAGCCACCCC

Supplementary Table 3. RT-qPCR primers used in this study

Kdm2b-JmjC-qPCR-F	AACTTGGCTTTGTACGAGGAGT
Kdm2b-JmjC-qPCR-R	CACGTTGAAGCTATGCAGGATG
Kdm2b-CxxC-qPCR-F	GTGGAGAGTGCCACTTTTGC
Kdm2b-CxxC-qPCR-R	CACTGTGTCCTCCTTCCCTG

Kdm2b-LRR-qPCR-F	GCTCATGGATCGCTGTCTCA
Kdm2b-LRR-qPCR-R	AGGCGCAGCTCTACAATGTT
Wnt7b-qPCR-F	GCTGGTCTCCGTCTATTGCC
Wnt7b-qPCR-R	TCACAATGATGGCATCGGGT
Wnt3-qPCR-F	GGGCCAGCAGTACACATCTCT
Wnt3-qPCR-R	CAGGCTGTCATCTATGGTGGT
Wnt10a-qPCR-F	TGAACACCCGGCCATACTTC
Wnt10a-qPCR-R	CATGTTCTCCATCACCGCCT
SFRP2-qPCR-F	GAAGAAATCCGTGCTGTGGC
SFRP2-qPCR-R	TGCGCTTGAACTCTCTCTGG
Wnt8b-qPCR-F	CCCGTGTGCGTTCTTCTAGT
Wnt8b-qPCR-R	CAACGGTCCCAAGCAAACTG
Wnt5a-qPCR-F	GTGATGCAAATAGGCAGCCG
Wnt5a-qPCR-R	AGCGTGGATTCGTTCCCTTT
Wnt5b-qPCR-F	GTGCCAACACCAGTTTCGAC
Wnt5b-qPCR-R	CTCTCGGGCATCCACAAACT
Wnt9a-qPCR-F	AACAACCTCGTGGGTGTGAAG
Wnt9a-qPCR-R	CTCTCCAGTGGCTTCATTGGT
Wnt3a-qPCR-F	TCTGCCATGAACCGTCACAA
Wnt3a-qPCR-R	GTACGTGTAACGTGGCCTCA
Wnt6-qPCR-F	GAGACGATGTGGACTTCGGG
Wnt6-qPCR-R	AGCCCATGGCACTTACACTC

Supplementary Table 4. Plasmid construction primers used in this study

Wnt3a-F-EcoR1	CATCATTTTGGCAAAGAATTCGCCACCATGGCTCCTC TCGGATACCTCTTAGTGCTC
Wnt3a-R-Not1	ATGGTCTTTGTAGTCGCGGCCGCCCTTGCAGGTGTG CACGTCATAGACAC
Wnt5a-F-EcoR1	CATCATTTTGGCAAAGAATTCGCCACCATGAAGAAGC CCATTGGAATATTAAGCCCGG
Wnt5a-R-Not1	ATGGTCTTTGTAGTCGCGGCCGCCTTTGCACACGAA CTGATCCACAATCTCC
Wnt5b-F-EcoR1	CATCATTTTGGCAAAGAATTCGCCACCATGCCCAGC CTGCTGCTGGTGGTCGTGGCA
Wnt5b-R-Not1	ATGGTCTTTGTAGTCGCGGCCGCCCTTACAGACATA CTGGTCCACAACCTCGGT
Wnt7b-F-EcoR1	CATCATTTTGGCAAAGAATTCGCCACCATGCACAGAA ACTTTCGAAAGTGGATCTTTT
Wnt7b-R-Kpn1	ATGGTCTTTGTAGTCGGTACCTCACTTGCAGGTGAA GACCTCGG
Wnt8b-F-EcoR1	CATCATTTTGGCAAAGAATTCGCCACCATGCTTCCCA TCTCTCAATGTTTGAGTCGC
Wnt8b-R-Kpn1	ATGGTCTTTGTAGTCGGTACCTTAGGAGTTCTTTCCC

	GGTTTGTG
SFRP2-F-EcoR1	CATCATTTTGGCAAAGAATTCGCCACCATGCCGCGG GGCCCTGCCTCGC
SFRP2-R-Kpn1	ATGGTCTTTGTAGTCGGTACCCTAGCATTGCAGCTTG CGGATGCTG

Supplementary Methods

Mice and genotyping

Mice with conditional deletion of *Kdm2b-CxxC* were obtained by first crossing *Kdm2b*$^{fl/fl}$ (generated by Applied Stem Cell) females with *Emx1-Cre* (Jackson Laboratories, stock number 005628), *Nestin-Cre* (Jackson Laboratories, stock number 003771) or *Nex-Cre* males [*Neurod6*$^{tm1(cre)Kan}$, MGI:2668659]. *Emx1-Cre; Kdm2b*$^{fl/+}$, *Nestin-Cre; Kdm2b*$^{fl/+}$ or *Nex-Cre; Kdm2b*$^{fl/+}$ males were crossed with *Kdm2b*^{flfl} females to obtain conditional knockout mice (*Kdm2b*$^{Emx1\text{-}\Delta CxxC}$, *Kdm2b*$^{Nestin\text{-}\Delta CxxC}$, *Kdm2b*$^{Nex\text{-}\Delta CxxC}$). *Kdm2b*$^{fl/+}$ and *Kdm2b*$^{fl/fl}$ were phenotypically indistinguishable from each other, and used as controls. The primer set forward 5'- cctgtagtccttggtatttcctggc-3'/reverse 5' - cccaacttgcccttaggccg-3' was used for mice genotyping, and band sizes for *Kdm2b*$^{fl/+}$ mice are 364 bp (WT allele) and 404 bp (targeted allele with 5' loxP). Forward 5'- cctgttacgtatagccgaaa-3'/reverse 5'- cttagcgccgtaaatcaatc-3' was used for *Emx1-Cre*, *Nestin-Cre* and *Nex-Cre* genotyping with band size 319 bp (Cre allele).

To analyze adult neurogenesis, the *Nestin-CreERT2* (Jackson Laboratories, stock number 016261) and *Kdm2b*$^{fl/fl}$ mice were crossed to generate *Nestin-CreERT2; Kdm2b*$^{fl/+}$ animals. *Nestin-CreERT2; Kdm2b*$^{fl/+}$ mice were further crossed with *Kdm2b*$^{fl/fl}$ mice to obtain homozygous *Kdm2b*$^{NestinCreERT2\text{-}\Delta CxxC}$ animals, which were used for the experiment. Forward 5'- gaccaggttcgttcactca-3'/reverse 5'- caagttaggagcaaacagtagc-3' was used for *Nestin-CreERT2* genotyping with band size 993 bp (CreERT2 allele).

To verify the activity of *Nestin-CreERT2* in hippocampal SGZ and the expression profile of *Emx1-Cre* and *Nestin-Cre* in developing hippocampus, we constructed *Nestin-CreERT2;Ai14* (Rosa-CAG-LSL-tdTomato-WPRE), *Emx1-Cre;Ai14* and *Nestin-Cre;Ai14* mice. The primer sets forward 5'- aagggagctgcagtggagta-3'/reverse 5' - ccgaaaatctgtgggaagtc-3' and forward 5'- ggcattaaagcagcgtatcc-3'/reverse 5' - ctgttcctgtacggcatgg-3' were used for *Ai14* mice genotyping, and band sizes for *Ai14+/-* mice are 297 bp (WT allele) and 196 bp (*Ai14* allele).

BAT-Gal mice were kind gifts from Dr. Junlei Chang (Jackson Lab, stock number 005317). To explore the Wnt signaling pathway in $Kdm2b^{Emx1\text{-}\Delta CxxC}$ mice, the BAT-Gal and $Kdm2b^{fl/fl}$ mice were crossed to generate $Kdm2b^{fl/+}$; BAT-Gal animals. *Emx1-Cre;* $Kdm2b^{fl/+}$ mice were further crossed with $Kdm2b^{fl/+}$; BAT-Gal mice to obtain $Kdm2b^{Emx1\text{-}\Delta CxxC}$; BAT-Gal animals, which were used for the experiment. The primer set forward 5′-atcctctgcatggtcaggtc-3′/reverse 5′-cgtggcctgattcattcc-3′ was used for BAT-Gal mice with band size 315 bp (LacZ allele).

Mice with conditional deletion of *Rnf2* were obtained by first crossing $Rnf2^{fl/fl}$ (purchased from GemPharmatech, Strain NO. T014803) females with *Emx1-Cre* males (Jackson Laboratories, stock number 005628). The primer set forward 5'- agctgtggtcctgcgtttcatttc-3'/reverse 5' - gctcttactgtgttacaaccctagccc-3' was used for $Rnf2^{fl/+}$ mice genotyping, and band sizes for $Rnf2^{fl/+}$ mice are 289 bp (WT allele) and 391 bp (targeted allele with 5' loxP).

Behavior tests

We used 12- to 16-week-old age-matched male mice for all behavioral tests. Mice were housed (3-5 animals per cage) in standard filter-top cages

with access to water and rodent chow at all times, maintained on a 12:12 h light/dark cycle (09:00-21:00 h lighting) at 22°C, with relative humidity of 50%–60%. All behavioral assays were done blind to genotypes.

Open field test. The test mouse was gently placed near the wall-side of a length of 50 cm, a width of 50 cm, and a height of 50 cm open-field arena and allowed to explore freely for 20 min. Only the last 10 min of the movement of the mouse was recorded by a video camera and analyzed with Ethovision XT 13 (Noldus).

Rotarod test. The test consists of 4 trials per day for 10 days with the rotarod (3 cm in diameter) set to accelerate from 4 rpm to 40 rpm over 5 minutes. The trial started once mice were placed on the rotarod rotating at 4 rpm in partitioned compartments. The time for each mouse spent on the rotarod were recorded. At least 20 min recovery time was allowed between trials. The rotarod apparatus was cleaned with 70% ethanol and wiped with paper towels between each trial.

Morris water maze. Mice were introduced into a stainless water-filled circular tank, which is 122 cm in diameter and 51 cm in height with non-reflective interior surfaces and ample visual cues. Two principal axes were drawn on the floor of the tank, each line bisecting the maze perpendicular to one another to create an imaginary '+'. The end of each line demarcates four cardinal points: North, South, East and West. To enhance the signal-to-noise ratio, the tank was filled with water colored with powdered milk. A 10-cm circular plexiglass platform was submerged 1 cm below the surface of the water in middle of the southwest quadrant. Mice started the task at fixed points, varied by day of testing[2]. Four trials were performed per mouse per day with 20 min intervals for 5 days. Each trial lasted 1 min and ended when the mouse climbed onto and remained on the hidden platform for 10 s. The mouse was given 20 s rest

on the platform during the inter-trial interval. The time taken by the mouse to reach the platform was recorded as its latency. Times for four trials were averaged and recorded as a result for each mouse. On day 6, the mouse was subjected to a single 60-s probe trial without a platform to test memory retention. The mouse started the trial from northeast, the number of platform crossings was counted, and the swimming path was recorded and analyzed using the Ethovision XT 13 (Noldus).

Fear conditioning (FC). The FC apparatus consisted of a conditioning box (18 × 18 × 30 cm), with a grid floor wired to a shock generator surrounded by an acoustic chamber and controlled by Ethovision XT 13 (Noldus). On Day 1, each mouse was placed in the conditioning box for 2 min, and then a pure tone (80 db) was sounded for 30 s followed by a 2 s foot shock (0.4 mA). Two minutes later, this procedure was repeated. After the delivery of the second shock, mice were returned to their home cages. On Day 2, each mouse was first placed in the fear conditioning chamber containing the exact same context, but there was no administration of a tone or foot shock. Freezing was analyzed for 4 min. One hour later, the mice were placed in a new context (containing a different odor, cleaning solution, floor texture, walls and shape) where they were allowed to explore for 3 min before being re-exposed to the fear conditioning tone and freezing was assessed for an additional 3 min. FC was assessed through the continuous measurement of freezing (complete immobility), which is the dominant behavioral fear response. Freezing was measured using the Noldus Ethovision video tracking system (Ethovision XT 13).

Forced swimming test. For the forced swimming test, the test mouse was placed into a 20 cm height and 17 cm diameter glass cylinder filled with water to a depth of 10 cm at 22°C. The test continues 6 min and the immobility time of the last 5 minutes was recorded for further processing.

Tail suspension test. The test mouse was suspended in the middle of a tail suspension box (55 cm height × 60 cm width × 11.5 cm depth) above the ground by its tail. The mouse tail was adhered securely to the suspension bar using adhesive tapes. After 1 min accommodation, the immobility time was recorded by a video camera and analyzed by Ethovision XT 13 (Noldus).

Elevated plus maze test. The elevated plus maze, made of gray polypropylene and elevated about 40 cm above the ground, consists of two open arms and two closed arms (each 9.5 cm wide and 40 cm long). To assess anxiety, the test mouse was placed in the central square facing an open arm and allowed to explore freely for 5 min. The time spent in the open arm was analyzed with the Ethovision XT 13 (Noldus).

Source Data

Source Data of Supplementary Fig 1b

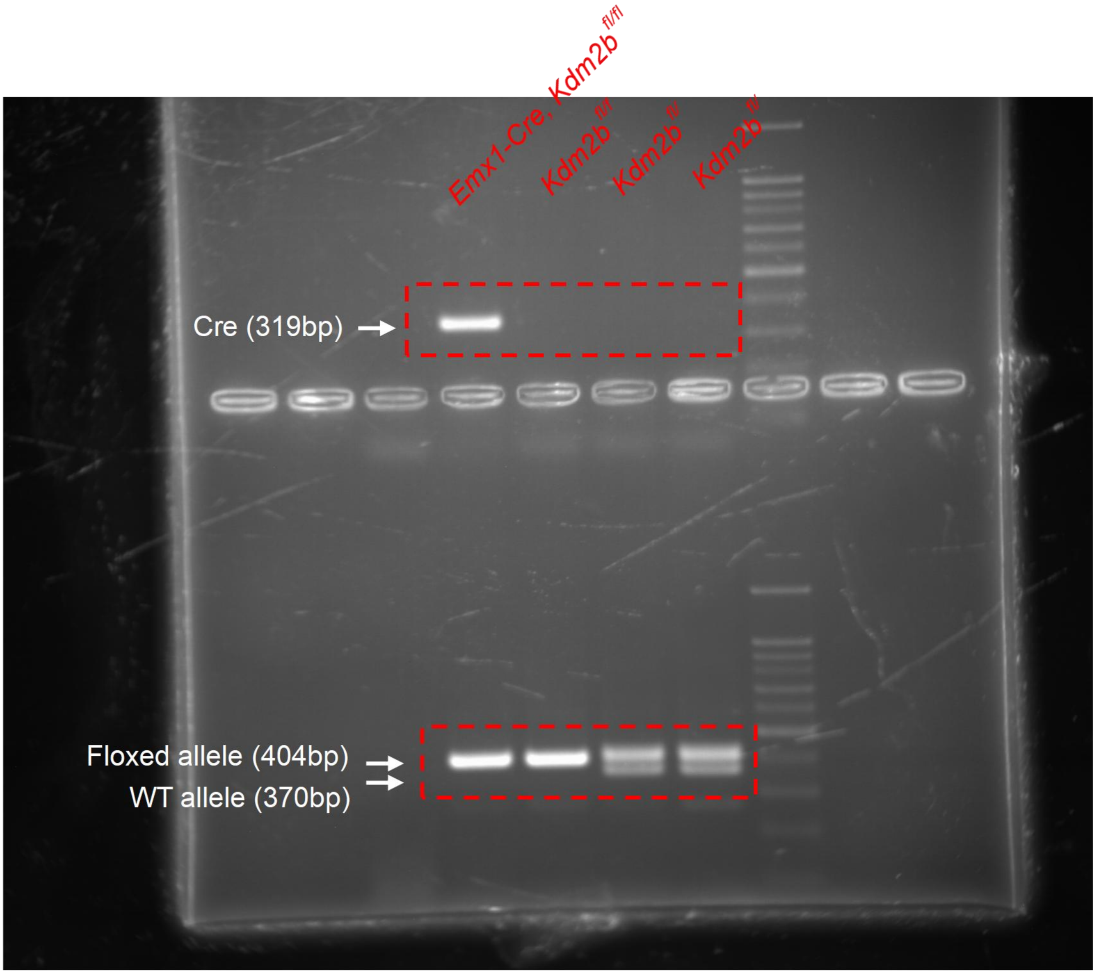

Source Data of Supplementary Fig 1k

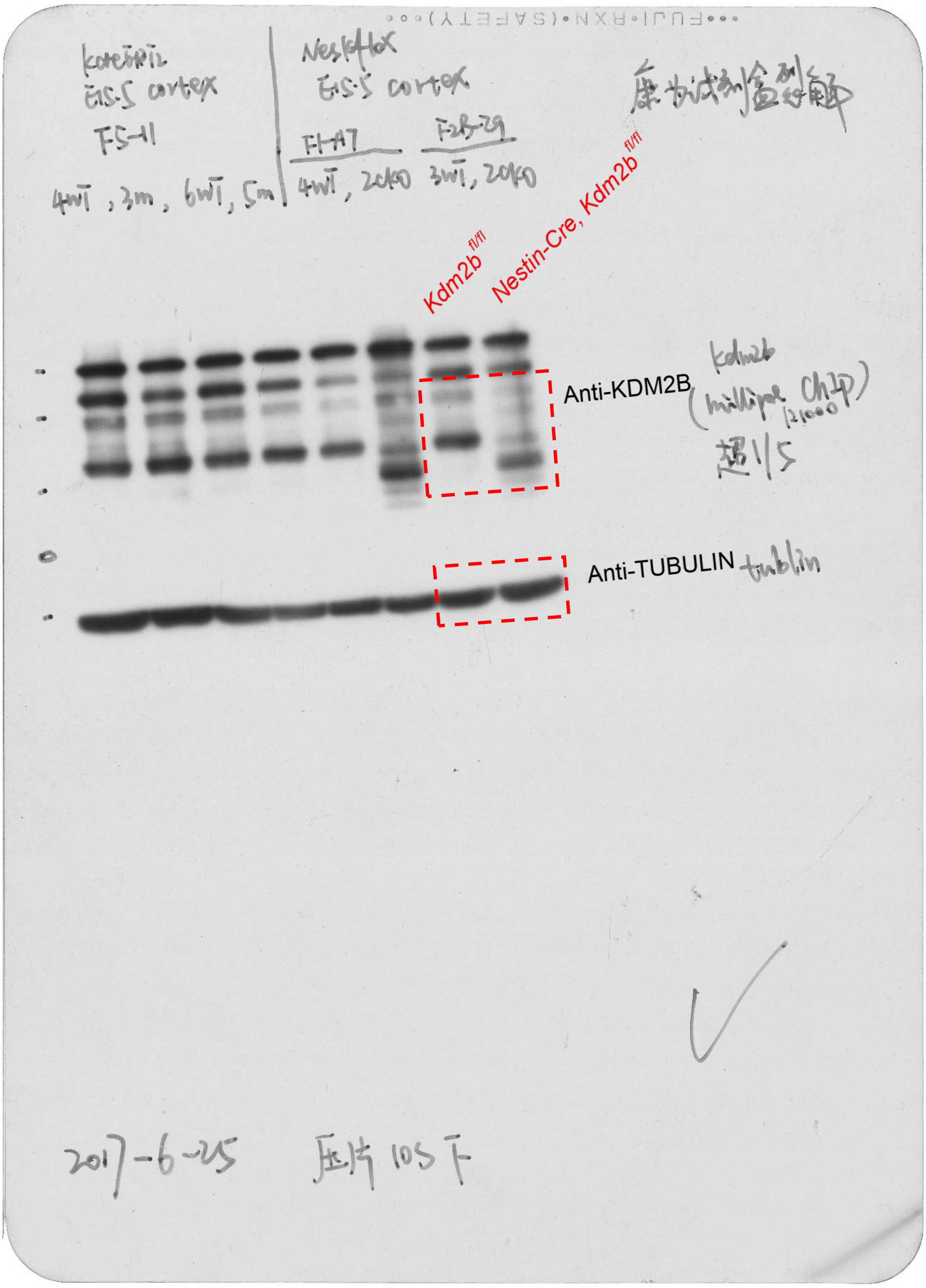

Source Data of Supplementary Fig 1l

Anti-KDM2B

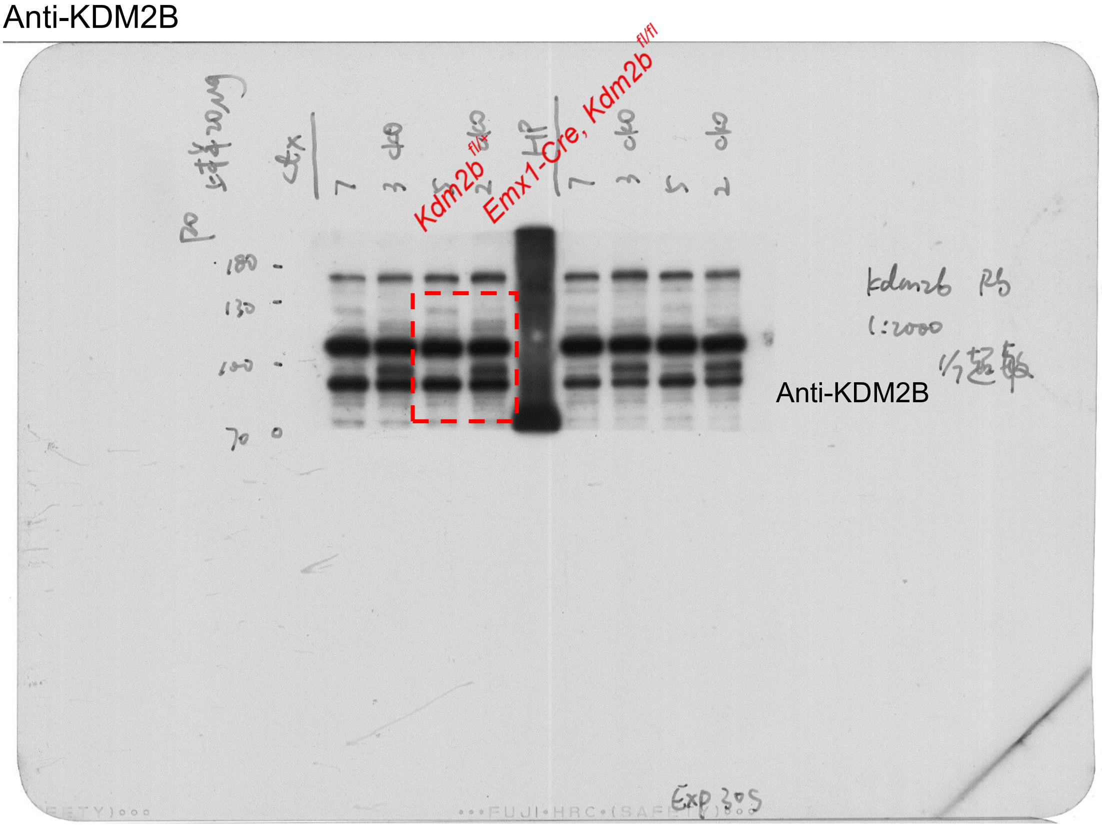

Anti-TUBULIN

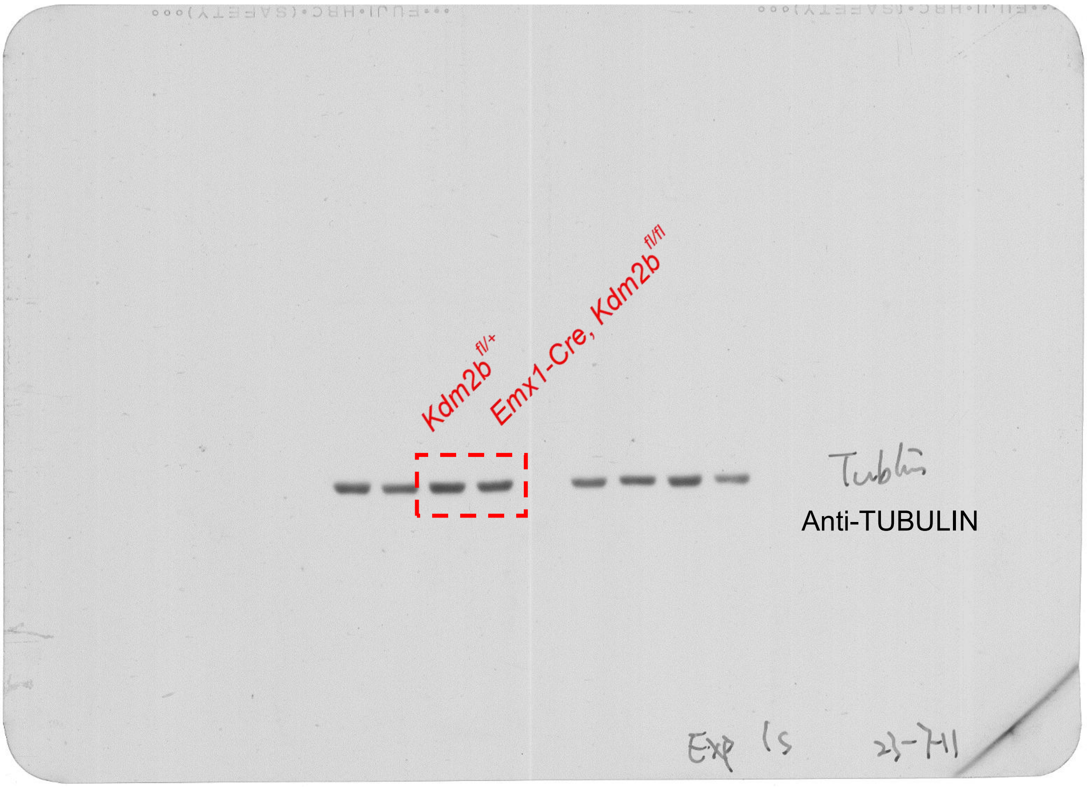

Source Data of Supplementary Fig 11i

Anti-H2AK119ub

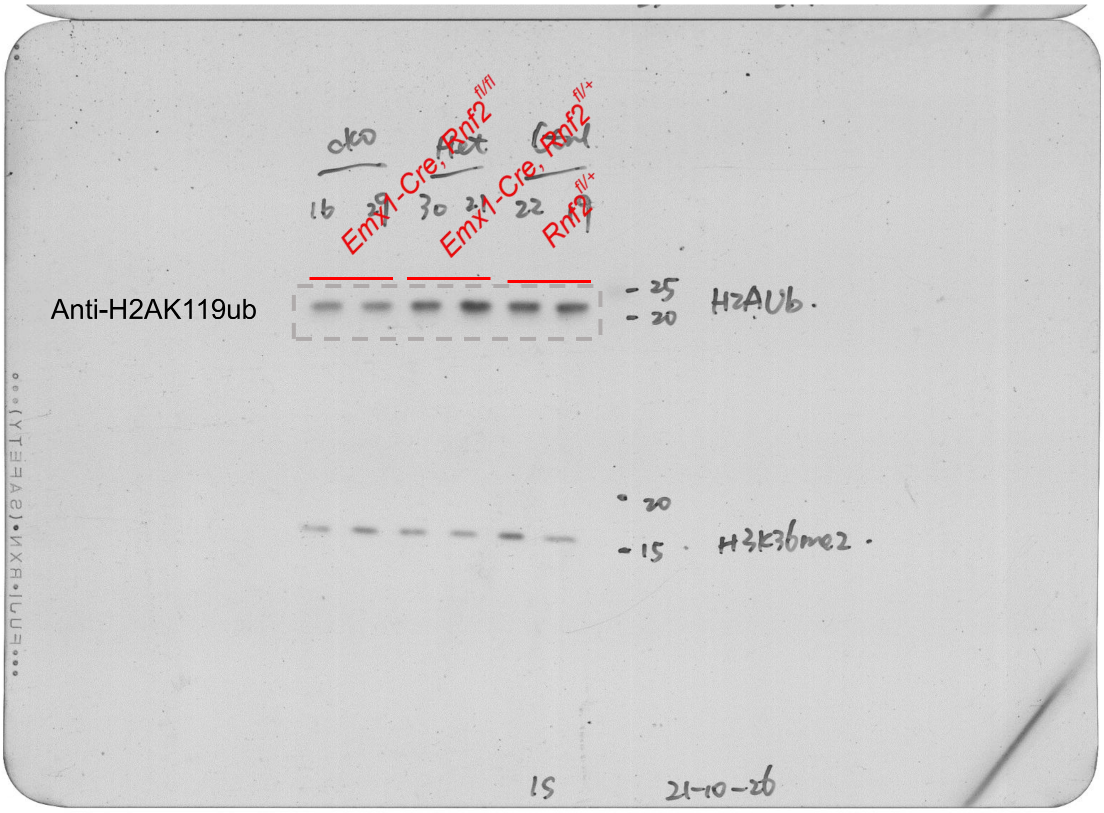

Anti-H3K27me3

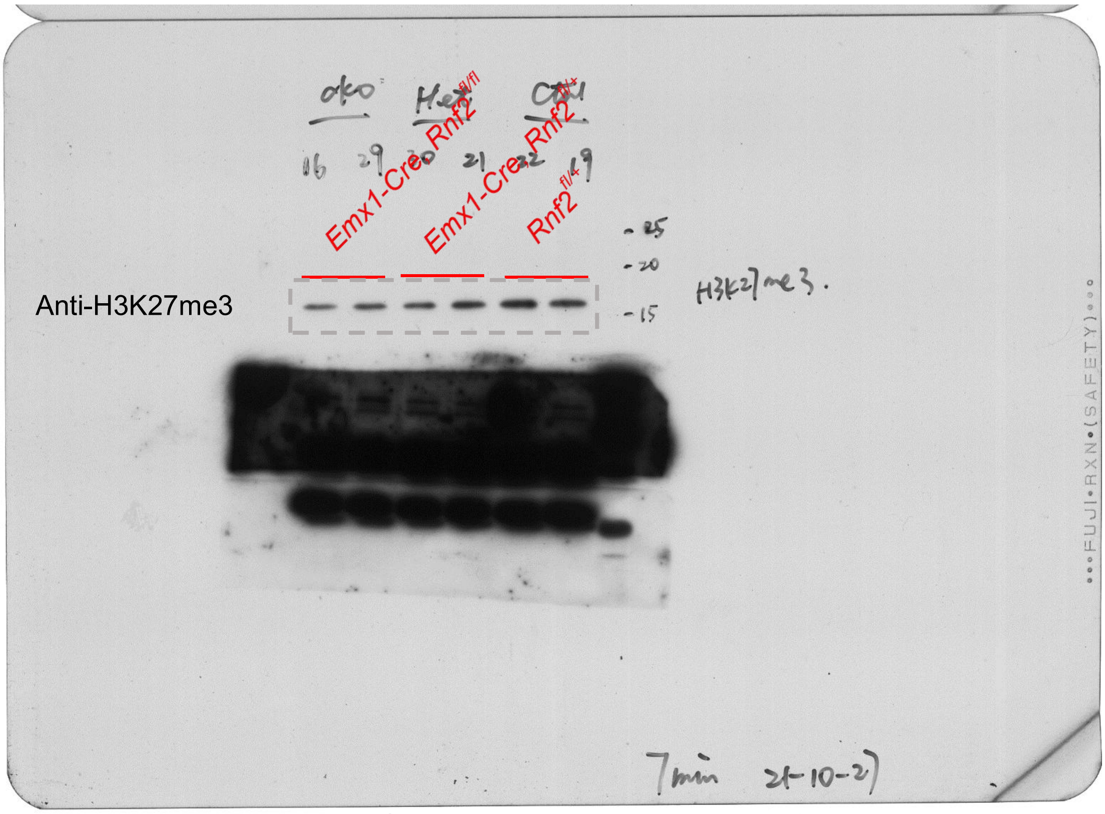

Anti-H3K36me2

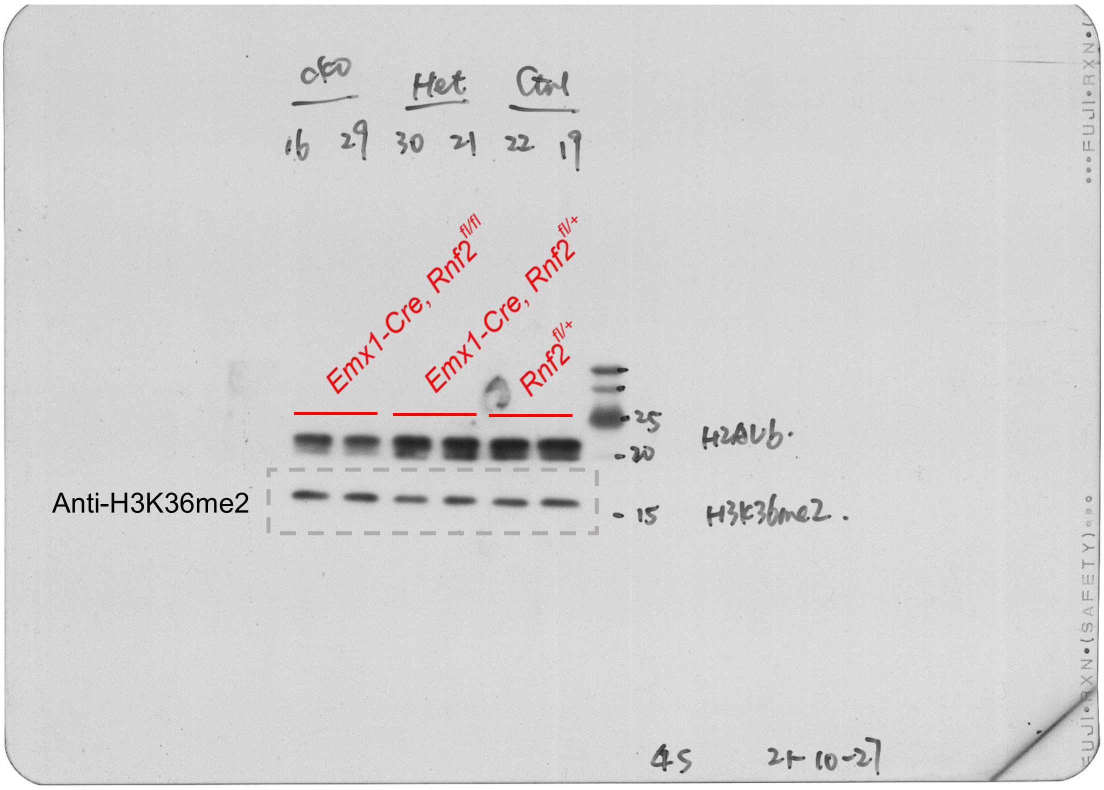

Anti-H3

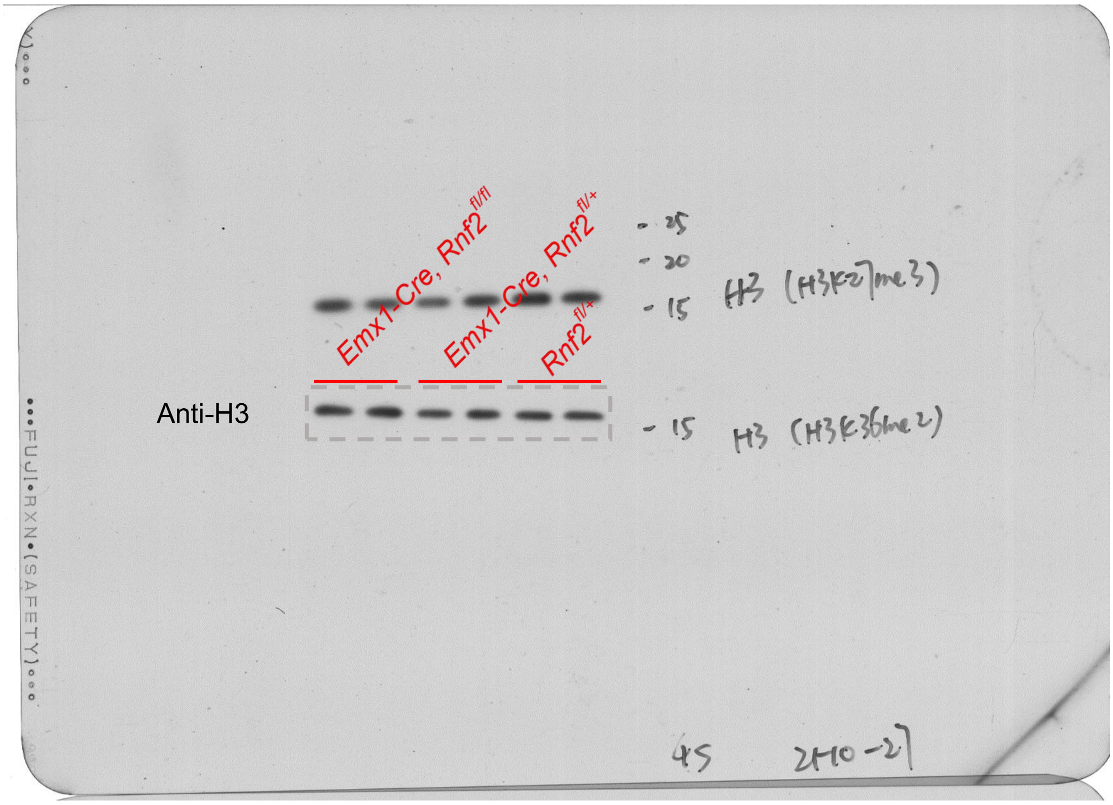

Supplementary References

1. Caramello A, Galichet C, Rizzoti K, Lovell-Badge R. Dentate gyrus development requires a cortical hem-derived astrocytic scaffold. *Elife* **10**, (2021).

2. Vorhees CV, Williams MT. Morris water maze: procedures for assessing spatial and related forms of learning and memory. *Nat Protoc* **1**, 848-858 (2006).
