# Supplementary material for: KDM2B regulates hippocampal morphogenesis by transcriptionally silencing Wnt signaling in neural progenitors
Source: Nat Commun. 2023 Oct 14;14:6489. doi: 10.1038/s41467-023-42322-2 (PMC10576813; doi:10.1038/s41467-023-42322-2)
Supplement: Supplementary file 3 — Reporting Summary [file 41467_2023_42322_MOESM3_ESM.pdf]

nature portfolio

Corresponding author(s): Yan Zhou

Last updated by author(s): Sep 26, 2023

Reporting Summary

Nature Portfolio wishes to improve the reproducibility of the work that we publish. This form provides structure for consistency and transparency in reporting. For further information on Nature Portfolio policies, see our Editorial Policies and the Editorial Policy Checklist.

Statistics

For all statistical analyses, confirm that the following items are present in the figure legend, table legend, main text, or Methods section.

n/a	Confirmed	
☐	☒	The exact sample size (*n*) for each experimental group/condition, given as a discrete number and unit of measurement
☐	☒	A statement on whether measurements were taken from distinct samples or whether the same sample was measured repeatedly
☐	☒	The statistical test(s) used AND whether they are one- or two-sided<br>*Only common tests should be described solely by name; describe more complex techniques in the Methods section.*
☒	☐	A description of all covariates tested
☐	☒	A description of any assumptions or corrections, such as tests of normality and adjustment for multiple comparisons
☐	☒	A full description of the statistical parameters including central tendency (e.g. means) or other basic estimates (e.g. regression coefficient) AND variation (e.g. standard deviation) or associated estimates of uncertainty (e.g. confidence intervals)
☐	☒	For null hypothesis testing, the test statistic (e.g. *F*, *t*, *r*) with confidence intervals, effect sizes, degrees of freedom and *P* value noted<br>*Give P values as exact values whenever suitable.*
☒	☐	For Bayesian analysis, information on the choice of priors and Markov chain Monte Carlo settings
☒	☐	For hierarchical and complex designs, identification of the appropriate level for tests and full reporting of outcomes
☒	☐	Estimates of effect sizes (e.g. Cohen's *d*, Pearson's *r*), indicating how they were calculated

Our web collection on statistics for biologists contains articles on many of the points above.

Software and code

Policy information about availability of computer code

Data collection	Immunohistochemical and immunofluorescence staining data were collected by ImageScope (x64), ZEN (version 2.3) and Leica Application Suite X (LAS X). Quantitative data for image processing were collected in ImageJ (version 1.8.0).
Data analysis	FastQC (version 0.11.9), cutadapt (version 3.2), STAR (version 2.7.10b), DESeq2 (version 1.38.1), Cufflinks package (version 2.2.1), clusterProfiler (version 4.2.2), Bowtie2 (version 2.4.5), SICER, Deeptools (version 3.5.1), MarkDuplicates (version 2.26.4), Macs2 (version 2.2.7.1), GraphPad Prism (version 8.0.2)

For manuscripts utilizing custom algorithms or software that are central to the research but not yet described in published literature, software must be made available to editors and reviewers. We strongly encourage code deposition in a community repository (e.g. GitHub). See the Nature Portfolio guidelines for submitting code & software for further information.

Data

Policy information about availability of data

All manuscripts must include a data availability statement. This statement should provide the following information, where applicable:

- Accession codes, unique identifiers, or web links for publicly available datasets
- A description of any restrictions on data availability
- For clinical datasets or third party data, please ensure that the statement adheres to our policy

The RNA-seq, ChIP-seq and ATAC-seq data generated in this study have been deposited in the NCBI GEO database under accession GSE222465 [https://www.ncbi.nlm.nih.gov/geo/query/acc.cgi?acc=GSE222465]. RNA-seq data of P0 hippocampus, P0 neurosphere and P0 cortex have been deposited at GEO:

GSE222464 [https://www.ncbi.nlm.nih.gov/geo/query/acc.cgi?acc=GSE222464]. ChIP-seq and ATAC-seq data of P0 hippocampus and P0 neurosphere in this study have been deposited at GEO: GSE222463 [https://www.ncbi.nlm.nih.gov/geo/query/acc.cgi?acc=GSE222463] and GSE222462 [https://www.ncbi.nlm.nih.gov/geo/query/acc.cgi?acc=GSE222462].

Research involving human participants, their data, or biological material

Policy information about studies with human participants or human data. See also policy information about sex, gender (identity/presentation), and sexual orientation and race, ethnicity and racism.

Reporting on sex and gender	N/A
Reporting on race, ethnicity, or other socially relevant groupings	N/A
Population characteristics	N/A
Recruitment	N/A
Ethics oversight	N/A

Note that full information on the approval of the study protocol must also be provided in the manuscript.

Field-specific reporting

Please select the one below that is the best fit for your research. If you are not sure, read the appropriate sections before making your selection.

[x] Life sciences [ ] Behavioural & social sciences [ ] Ecological, evolutionary & environmental sciences

For a reference copy of the document with all sections, see nature.com/documents/nr-reporting-summary-flat.pdf

Life sciences study design

All studies must disclose on these points even when the disclosure is negative.

Sample size	Animal numbers were determined by pilot data and according to previous studies in our laboratory (PMID: 31317506, 33523829).
Data exclusions	No data was excluded from analyses in this study.
Replication	For all experiment, each replicate indicates a distinct mouse sample, and it was described in the figure legends and methods.<br>At least 3-5 biological replicates for phenotypic analysis;<br>At least 10 biological replicates were used in mouse behavioral experiments;<br>At least 2 biological replicates were used for high-throughput sequencing;<br>At least 5 biological replicates were used in IUE experiments.
Randomization	The mice were divided into control group and cKO group according to genotype. Within each group, mice were selected randomly.
Blinding	All animal treatments, collections, and data analyses were performed in a blinded fashion.

Reporting for specific materials, systems and methods

We require information from authors about some types of materials, experimental systems and methods used in many studies. Here, indicate whether each material, system or method listed is relevant to your study. If you are not sure if a list item applies to your research, read the appropriate section before selecting a response.

Materials & experimental systems

n/a	Involved in the study	
[ ]	[x]	Antibodies
[x]	[ ]	Eukaryotic cell lines
[x]	[ ]	Palaeontology and archaeology
[ ]	[x]	Animals and other organisms
[x]	[ ]	Clinical data
[x]	[ ]	Dual use research of concern
[x]	[ ]	Plants

Methods

n/a	Involved in the study	
[ ]	[x]	ChIP-seq
[x]	[ ]	Flow cytometry
[x]	[ ]	MRI-based neuroimaging

Antibodies

Antibodies used	primary antibodies:<br>mouse anti-Calbindin (1:1000; Sigma, C9848, clone CB-955),<br>rabbit anti-ZBTB20 (1:1000; Sigma, HPA016815),<br>mouse anti-HopX (1:200; Santa Cruz, sc-398703, E-1),<br>rabbit anti-Wfs1 (1:1000; Proteintech, 26995-1-AP, 86995),<br>mouse anti-PROX1 (1:200; Millipore, MAB5654, clone 4G10),<br>rabbit anti-GFAP (1:500; DAKO, Z0334),<br>rat anti-CTIP2 (1:500; Abcam, ab18465, 25B6),<br>rabbit anti-SATB2 (1:500; Abcam, ab92446, EPNCIR130A),<br>rat anti-BrdU (1:500; Abcam, ab6326, BU1/75 (ICR1)),<br>mouse-anti-BrdU (1:500; Roche, 11170376001),<br>rabbit-anti-DCX (1:500; Abcam, ab18723),<br>rabbit anti-TBR2 (1:500; Abcam, ab23345),<br>rat anti-TBR2 (1:500; Thermo Fisher, 14-4875-82, Dan11mag),<br>rabbit anti-PAX6 (1:500; Millipore, ab2237),<br>rabbit anti-Ki67 (1:500; Abcam, ab15580)<br>rabbit anti-NeuN (1:500; Abcam, ab177487, EPR12763),<br>rabbit anti-SOX2 (1:500; Millipore, ab5603),<br>rabbit anti-BLBP (1:500; Abcam, ab32423),<br><br>secondary antibodies:<br>Alexa Fluor 488-conjugated anti-mouse, A11029; Thermo Fisher Scientific; 1:1000;<br>Alexa Fluor 555-conjugated anti-mouse, A21422; Thermo Fisher Scientific; 1:1000;<br>Alexa Fluor 488-conjugated anti-rat, A11006;Thermo Fisher Scientific; 1:1000;<br>Alexa Fluor 555-conjugated anti-rat, A21434; Thermo Fisher Scientific; 1:1000;<br>Alexa Fluor 647-conjugated anti-rat, A21247; Thermo Fisher Scientific; 1:1000;<br>Alexa Fluor 488-conjugated anti-rabbit, A11034; Thermo Fisher Scientific; 1:1000;<br>Alexa Fluor 555-conjugated anti-rabbit, A21429; Thermo Fisher Scientific; 1:1000;<br>Alexa Fluor 647-conjugated anti-rabbit, A21245; Thermo Fisher Scientific; 1:1000;<br><br>Primary antibodies for ChIP-seq:<br>rabbit anti-H2AK119Ub antibody (CST, 8240S, D27C4),<br>rabbit anti-H3K27me3 antibody (CST, 9733S, C36B11),<br>rabbit anti-H3K36me2 antibody (CST, 2901S, C75H12).
Validation	All used antibodies are IF and ChIP grade and were validated by manufacturer. We also conducted IF and ChIP-qPCR verification before using antibodies in formal experiments.

Animals and other research organisms

Policy information about studies involving animals; ARRIVE guidelines recommended for reporting animal research, and Sex and Gender in Research

Laboratory animals	Kdm2b-cKO (Kdm2bEmx1-ΔCxxC, Kdm2bNestin-ΔCxxC, Kdm2bNex-ΔCxxC) or control(Kdm2bfl/+ and Kdm2bfl/fl) mice were collected at various stages of hippocampal development (E13.5, E14.5, E16.5, E18.5, P0, P2, P7, adult, etc.).<br>Emx1-Cre;Ai14(Rosa-CAG-LSL-tdTomato-WPRE) mice were collected at P0, and Nestin-Cre;Ai14 mice were collected at E13.5.<br>Nestin-CreERT2;Ai14 were collected at adulthood (10 weeks and 13 weeks).<br>Nestin-CreERT2; Kdm2bfl/fl mice were collected at adulthood (10 weeks and 13 weeks).<br>Kdm2bEmx1-ΔCxxC; BAT-Gal were collected at P0.<br>Rnf2Emx1-cKO were collected at P0.<br>Wild-type CD-1 (ICR) mice were collected at E18.5 for IUE.
Wild animals	No wild animals were used.
Reporting on sex	For phenotypic analyses of hippocampal development at embryonic and postnatal stages, we did not consider sex difference. The Kdm2b gene is not located on the sex chromosome and has not been reported to be associated with sex determination.<br>For adult behavioral experiments, male mice were selected according to the general standard.
Field-collected samples	No field-collected samples were used in this study.
Ethics oversight	All experimental procedures were approved by the Animal Care and Ethical Committee of Medical Research Institute, Wuhan University. Informed consent was obtained from all participants of the study.

Note that full information on the approval of the study protocol must also be provided in the manuscript.

Plants

Seed stocks	*Report on the source of all seed stocks or other plant material used. If applicable, state the seed stock centre and catalogue number. If plant specimens were collected from the field, describe the collection location, date and sampling procedures.*
Novel plant genotypes	*Describe the methods by which all novel plant genotypes were produced. This includes those generated by transgenic approaches, gene editing, chemical/radiation-based mutagenesis and hybridization. For transgenic lines, describe the transformation method, the number of independent lines analyzed and the generation upon which experiments were performed. For gene-edited lines, describe the editor used, the endogenous sequence targeted for editing, the targeting guide RNA sequence (if applicable) and how the editor was applied.*
Authentication	*Describe any authentication procedures for each seed stock used or novel genotype generated. Describe any experiments used to assess the effect of a mutation and, where applicable, how potential secondary effects (e.g. second site T-DNA insertions, mosiacism, off-target gene editing) were examined.*

ChIP-seq

Data deposition

☒ Confirm that both raw and final processed data have been deposited in a public database such as GEO.

☒ Confirm that you have deposited or provided access to graph files (e.g. BED files) for the called peaks.

Data access links<br>*May remain private before publication.*	ChIP-seq data of P0 hippocampus and P0 neurosphere in this study have been deposited at GEO: GSE222463 [https://www.ncbi.nlm.nih.gov/geo/query/acc.cgi?acc=GSE222463].
Files in database submission	GSM6923712 Hippocampus, ATAC, cKO, rep1<br>GSM6923713 Hippocampus, ATAC, cKO, rep2<br>GSM6923714 Hippocampus, ATAC, Ctrl, rep1<br>GSM6923715 Hippocampus, ATAC, Ctrl, rep2<br>GSM6923716 P0-Hippocampus_cKO-H2AK119ub1_rep1<br>GSM6923717 P0-Hippocampus_cKO-H2AK119ub1_rep2<br>GSM6923718 P0-Hippocampus_cKO-H3K27me3_rep1<br>GSM6923719 P0-Hippocampus_cKO-H3K27me3_rep2<br>GSM6923720 P0-Hippocampus_cKO_H3K36me2_Input_rep1<br>GSM6923721 P0-Hippocampus_cKO_H3K36me2_Input_rep2<br>GSM6923722 P0-Hippocampus_cKO_H3K36me2_rep1<br>GSM6923723 P0-Hippocampus_cKO_H3K36me2_rep2<br>GSM6923724 P0-Hippocampus_cKO-Input_rep1<br>GSM6923725 P0-Hippocampus_cKO-Input_rep2<br>GSM6923726 P0-Hippocampus_Ctrl-H2AK119ub1_rep1<br>GSM6923727 P0-Hippocampus_Ctrl-H2AK119ub1_rep2<br>GSM6923728 P0-Hippocampus_Ctrl-H3K27me3_rep1<br>GSM6923729 P0-Hippocampus_Ctrl-H3K27me3_rep2<br>GSM6923730 P0-Hippocampus_Ctrl_H3K36me2_Input_rep1<br>GSM6923731 P0-Hippocampus_Ctrl_H3K36me2_Input_rep2<br>GSM6923732 P0-Hippocampus_Ctrl_H3K36me2_rep1<br>GSM6923733 P0-Hippocampus_Ctrl_H3K36me2_rep2<br>GSM6923734 P0-Hippocampus_Ctrl-Input_rep1<br>GSM6923735 P0-Hippocampus_Ctrl-Input_rep2<br>GSM6923736 P0-Neurosphere_cKO-H2AK119Ub1_rep1<br>GSM6923737 P0-Neurosphere_cKO-H2AK119Ub1_rep2<br>GSM6923738 P0-Neurosphere_cKO-H3K27me3_rep1<br>GSM6923739 P0-Neurosphere_cKO-H3K27me3_rep2<br>GSM6923740 P0-Neurosphere_cKO-H3K36me2_rep1<br>GSM6923741 P0-Neurosphere_cKO-H3K36me2_rep2<br>GSM6923742 P0-Neurosphere_cKO-Input_rep1<br>GSM6923743 P0-Neurosphere_cKO-Input_rep2<br>GSM6923744 P0-Neurosphere_Ctrl-H2AK119Ub1_rep1<br>GSM6923745 P0-Neurosphere_Ctrl-H2AK119Ub1_rep2<br>GSM6923746 P0-Neurosphere_Ctrl-H3K27me3_rep1<br>GSM6923747 P0-Neurosphere_Ctrl-H3K27me3_rep2<br>GSM6923748 P0-Neurosphere_Ctrl-H3K36me2_rep1<br>GSM6923749 P0-Neurosphere_Ctrl-H3K36me2_rep2<br>GSM6923750 P0-Neurosphere_Ctrl-Input_rep1<br>GSM6923751 P0-Neurosphere_Ctrl-Input_rep2<br>GSM6923752 Hippocampus, Ctrl, rep1<br>GSM6923753 Hippocampus, Ctrl, rep2<br>GSM6923754 Hippocampus, Ctrl, rep3

GSM6923755 Hippocampus, cKO, rep1
GSM6923756 Hippocampus, cKO, rep2
GSM6923757 Hippocampus, cKO, rep3
GSM6923758 Neurosphere, Ctrl, rep1
GSM6923759 Neurosphere, Ctrl, rep2
GSM6923760 Neurosphere, cKO, rep1
GSM6923761 Neurosphere, cKO, rep2
GSM7186908 P0_Cortex_Kdm2b_Ctrl_rep1
GSM7186909 P0_Cortex_Kdm2b_Ctrl_rep2
GSM7186910 P0_Cortex_Kdm2b_cKO_rep1
GSM7186911 P0_Cortex_Kdm2b_cKO_rep2
GSM7186912 P0_HP_Rnf2_Ctrl_rep1
GSM7186913 P0_HP_Rnf2_Ctrl_rep2
GSM7186914 P0_HP_Rnf2_cKO_rep1
GSM7186915 P0_HP_Rnf2_cKO_rep2

Genome browser session (e.g. UCSC)

http://genome.ucsc.edu/s/Bo1996/Zhang%202023

Methodology

Replicates

Two biological replicates were set up in each ChIP-seq group, and the antibodies were verified by ChIP-qPCR in advance.

Sequencing depth

20-35 million reads [150 base pair (bp) single reads] were obtained for each library.

Antibodies

rabbit anti-H2AK119Ub antibody (CST, 8240S)
rabbit anti-H3K27me3 antibody (CST, 9733S)
rabbit anti-H3K36me2 antibody (CST, 2901S)

Peak calling parameters

Regions of peaks were called using the SICER software package, with the input genomic DNA as a background control (parameters: -w 200 -rt 1 -f 150 -egf 0.77 -fdr 0.01 -g 600 -e 1000 --significant_reads)

Data quality

H2AK119ub1 (SICER FDR 0.01) total peaks: Ctrl_rep1(17364), Ctrl_rep2(26417), cKO_rep1(13711), cKO_rep2(53420);
H3K27me3 (SICER FDR 0.01) total peaks: Ctrl_rep1(27697), Ctrl_rep2(12205), cKO_rep1(23353), cKO_rep2(38356);
H3k36me2 (SICER FDR 0.01) total peaks: Ctrl_rep1(48314), Ctrl_rep2(40696), cKO_rep1(43074), cKO_rep2(31578)

Software

Bowtie2 (version 2.4.5), SICER, Deeptools (version 3.5.1), Homer
